# Dressing AgNWs with MXenes Nanosheets: Transparent Printed Electrodes Combining High‐Conductivity and Tunable Work Function for High‐Performance Opto‐Electronics

**DOI:** 10.1002/adma.202412512

**Published:** 2024-10-14

**Authors:** Zhongshi Ju, Yusheng Chen, Peng Li, Jiangang Ma, Haiyang Xu, Yichun Liu, Paolo Samorì

**Affiliations:** ^1^ Key Laboratory of UV‐Emitting Materials and Technology Ministry of Education Northeast Normal University Changchun 130024 P. R. China; ^2^ Université de Strasbourg, CNRS, ISIS 8 allée Gaspard Monge Strasbourg 67000 France

**Keywords:** gallium oxide, MXene, organic light‐emitting transistor, Schottky diode, transparent electrode, tunable work function

## Abstract

High‐work function transparent electrodes (HWFTEs) are key for establishing Schottky and Ohmic contacts with n‐type and p‐type semiconductors, respectively. However, the development of printable materials that combine high transmittance, low sheet resistance, and tunable work function remains an outstanding challenge. This work reports a high‐performance HWFTE composed of Ag nanowires enveloped conformally by Ti_3_C_2_T_x_ nanosheets (TA), forming a shell‐core network structure. The printed TA HWFTEs display an ultrahigh transmittance (>94%) from the deep‐ultraviolet (DUV) to the entire visible spectral region, a low sheet resistance (<15 Ω sq^−1^), and a tunable work function ranging from 4.7 to 6.0 eV. The introduction of additional oxygen terminations on the Ti_3_C_2_T_x_ surface generates positive dipoles, which not only increases the work function of the TA HWFTEs but also elevates the TA/Ga_2_O_3_ Schottky barrier, resulting in a high self‐powered responsivity of 18 mA W^−1^ in Ga_2_O_3_ diode DUV photodetectors, as demonstrated via experimental characterizations and theoretical calculations. Furthermore, the TA HWFTEs‐based organic light‐emitting transistors exhibit exceptional emission brightness of 5020 cd m^−2^, being four‐fold greater than that in Au electrodes‐based devices. The innovative nano‐structure design, work function tuning, and the revealed mechanisms of electrode‐semiconductor contact physics constitute a substantial advancement in high‐performance optoelectronic technology.

## Graphical abstract

Printable highly conductive UV–vis transparent Ti_3_C_2_T_x_‐AgNWs electrodes with tunable work functions are assembled by regulating the O‐related terminations on the surface of Ti_3_C_2_T_x_. The electrode can be customized for energy band matching, e.g. forming Schottky and Ohmic contacts with Ga_2_O_3_ and SpiroG in high‐performance Schottky diodes and organic light‐emitting transistors, respectively.

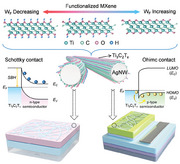

## 1 Introduction

Transparent electrodes are essential in optoelectronic devices as they enable, among others, light out‐coupling in light‐emitting devices and light absorption in photovoltaic devices.^[^ ^1^, ^2^ ^]^ The primary physical properties that need to be simultaneously optimized in transparent electrodes for their application in high‐performance devices include their work function, transmittance, and sheet resistance.^[^ ^3^ ^]^ Tailoring the work function of transparent electrodes via surface functionalization while maintaining their high full‐spectrum transmittance and low sheet resistance represents a powerful strategy to facilitate charge injection and extraction at electrode‐semiconductor interfaces by tuning the energy barrier in line with the energy levels of the chosen semiconductor. For example, increasing the work function of transparent electrodes can promote the establishment of Ohmic or Schottky contacts with p‐type organic semiconductors that possess low Fermi levels near their highest occupied molecular orbital (HOMO) and n‐type inorganic semiconductors with high Fermi levels near their conduction band minimum (CBM).^[^ ^4^, ^5^, ^6^ ^]^ This procedure is of paramount importance in the fabrication of high‐performance optoelectronic devices. Nonetheless, noble metals such as gold (Au) and platinum (Pt), which offer high work functions of ≈5.2 and 5.6 eV, respectively, exhibit a high level of optical reflectivity. This is attributed to the oscillating electric field of light waves, which stimulates an excess of free electrons within the metal, leading them to oscillate and subsequently re‐emit electromagnetic waves.^[^ ^7^ ^]^ In contrast, the commonly utilized transparent conductive materials, such as indium tin oxide (ITO), aluminum‐doped zinc oxide (AZO), and fluorine‐doped tin oxide (FTO), feature relatively lower work functions, typically ranging between 4.5 and 4.7 eV, which is detrimental to the formation of Ohmic contacts with p‐type organic semiconductors and Schottky contacts with n‐type inorganic semiconductors.

Recent efforts have been devoted to increasing the work function of transparent conductive oxides by surface functionalization.^[^ ^8^, ^9^, ^10^ ^]^ However, high‐work function transparent electrodes (HWFTEs) fabricated with these conventional materials and related processing methods suffer from a narrow adjustable range of work function and poor reproducibility, jeopardizing their use as top window electrodes in optoelectronic devices. Moreover, the pronounced band edge absorption and inherent fragility of transparent conductive oxides preclude their use in deep‐ultraviolet (DUV) optoelectronics and flexible electronics, further highlighting the need for the development of next‐generation HWFTEs.

As emerging 2D conductors, transition‐metal carbides and nitrides—such as Ti_3_C_2_T_x_ MXenes—boast a combination of solution‐processability, a large specific surface area, and intrinsic high conductivity,^[^ ^11^, ^12^, ^13^, ^14^, ^15^ ^]^ rendering them ideal electrode materials for a range of electronic devices.^[^ ^16^, ^17^, ^18^, ^19^, ^20^, ^21^, ^22^, ^23^, ^24^, ^25^, ^26^, ^27^ ^]^ Most significantly, the abundant surface terminations in MXenes offer the opportunity to fine‐tune their work function in an energy range exceeding 1.0 eV, which holds the potential to advance semiconductor technologies through the optimization of MXene‐semiconductor interfaces. Nevertheless, progress in developing MXenes HWFTE is slowed by the limited availability of effective techniques for customizing their surface terminations on demand and by the difficulty in striking a balance between high transmittance and low sheet resistance. As a result, despite its huge potential, the enhancement of optoelectronic device performance using MXenes HWFTEs is underexplored. The development of robust strategies for controlling MXenes’ surface terminations is expected to harness the full potential of MXenes HWFTEs. Furthermore, gaining a deeper insight into how the work function of MXenes is influenced by surface terminations, and the resultant effect on the energy band structure of neighboring semiconductors, is critical. This understanding has the potential to reveal intriguing physical phenomena that go beyond the traditional comprehension of metal‐semiconductor interactions.

Pursuing the challenge of finding solutions to assemble electrodes that combine high transmittance, low sheet resistance, and a tunable work function in printable transparent electrodes, the integration of silver nanowires (AgNWs) networks with MXene nanosheets emerges as a promising strategy. Specifically, the AgNWs networks, known for their high conductivity, full‐spectrum transparency, and mechanical flexibility, can act as a conductive scaffold. The MXene nanosheets can conformally envelop this scaffold, serving as the contact layer. This combination leverages the tunable work function of MXenes while alleviating their inherent intense absorption, especially in the DUV spectrum. However, beyond the essential requirement of effectively controlling the work function of MXenes, the assembling of the Ti_3_C_2_T_x_‐AgNWs (TA) shell‐core network is technically difficult yet crucial for ensuring their high transmittance in the DUV region. These unresolved challenges currently impede the development of high‐performance TA HWFTEs and limit their potential applications.

**Figure 1:**
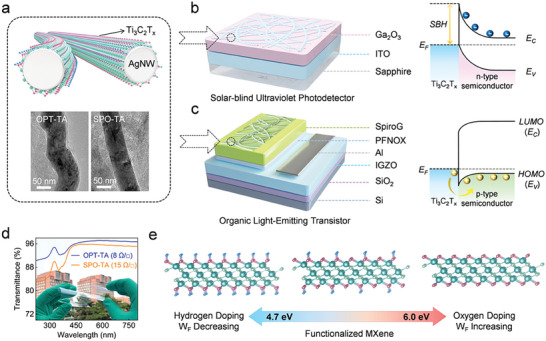
a) Schematic structure of hybrid formed by coating the AgNWs with Ti_3_C_2_T_x_ nanosheets and assembling them into networks. TEM image depicting the core‐shell structure of AgNWs‐Ti_3_C_2_T_x_. Schematic diagrams of the device structures of b) Schottky diode photodetector and c) light‐emitting transistor, where the TA forms the Schottky contact with n‐type inorganic semiconductor and the Ohmic contact with p‐type organic semiconductor, respectively. d) Transmission spectra of the TA network and TA film. The inset shows the photograph of the TA network and the TA film on the flexible substrates. e) Schematic of the Ti_3_C_2_T_x_ with tunable work function modified by oxygen and hydroxyl functional groups.

The present study developed unprecedented printable HWFTEs based on surface functionalized TA hybrid, comprising oxidized Ti_3_C_2_T_x_ contact layer as conformal coating of AgNWs conductive skeletons (**Figure** **1**a) which are assembled into networks. The oxidization of Ti_3_C_2_T_x_ was accomplished by means of either oxygen plasma treatment (OPT) or solution‐processed oxidation (SPO). The Ti_3_C_2_T_x_ nanosheets can be either conformally wrapped onto AgNWs forming a core–shell network (OPT‐TA) or directly placed underneath and above the AgNWs (SPO‐TA) to be readily exploited as transparent electrodes in DUV and visible optoelectronic devices (Figure 1b,c), respectively. The printed OPT‐TA network and SPO‐TA film electrodes combine a low sheet resistance of 8 and 15 Ω sq^−1^ with a DUV–vis spectrum average transmittance of 96% and 94%, respectively, as shown in Figure 1d. Kelvin probe force microscope (KPFM) analyses indicated that OPT‐oxidated dispersed Ti_3_C_2_T_x_ nanosheets and SPO‐oxidized Ti_3_C_2_T_x_ nanosheet hydrocolloids display adjustable work functions in the ultra‐wide range spanning from 4.7 to 6.0 eV, i.e., exceeding 1.0 eV. (Figure 1e). These functionalized TA HWFTEs are therefore suitable to engineer Schottky contact with n‐type inorganic semiconductors and Ohmic contact with p‐type organic semiconductors. As a proof‐of‐concept demonstration, the OPT‐TA networks were employed as transparent electrodes to fabricate TA/Ga_2_O_3_ Schottky diodes serving as DUV photodetectors. Additionally, the SPO‐TA films were utilized as the drain electrode in top‐emission organic light‐emitting transistors (OLETs).

Our experimental and theoretical studies have revealed: i) a significant increase in the work function of Ti_3_C_2_T_x_ upon increasing the O terminations which can be attributed to the enhanced positive surface dipole moment density; ii) an exceptionally high TA/Ga_2_O_3_ Schottky barrier height, exceeding the value predicted by the conventional Schottky‐Mott relation, which can be achieved due to the dipole‐induced elevation of the electron affinity. This enhancement can increase the built‐in field and responsivity of TA/Ga_2_O_3_ self‐powered DUV photodetectors; iii) an improvement in the emission brightness of OLETs with a TA anode compared to those with an Au film anode, by minimizing the energy level gap for hole transfer and reducing light loss. Ultimately, our printed TA hybrid‐based HWFTEs combine all the key characteristics required for boosting the performance of a large portfolio of electronic and optoelectronic devices.

## 2 Results and Discussion

### 2.1 Work Function Modulation of Ti_3_C_2_T_x_

**Figure 2:**
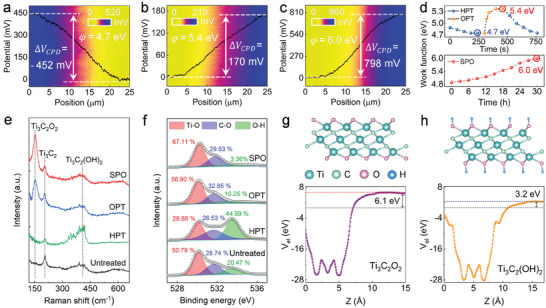
Surface potential distribution was recorded on the Ti_3_C_2_T_x_ films after a) hydrogen plasma treatment (HPT), b) oxygen plasma treatment (OPT), and c) solution‐processed oxidation (SPO), respectively. d) Variation of the work function of the Ti_3_C_2_T_x_ films with the alternate HPT and OPT time and variation of the work function of the Ti_3_C_2_T_x_ films with SPO time. e) Raman spectra and f) O 1s XPS spectra of the untreated, HPT, OPT, and SPO Ti_3_C_2_T_x_ films. DFT calculated structures (up panels) and plane‐averaged electrostatic potentials (V_el_) of g) Ti_3_C_2_O_2_ and h) Ti_3_C_2_(OH)_2_.

Two scalable methods were exploited to modify the work function of Ti_3_C_2_T_x_ hydrocolloids: i) oxygen plasma treatment to adjust the terminations of dispersed Ti_3_C_2_T_x_ nanosheets on the substrates, and ii) in situ thermal oxidization on the hydrocolloids to introduce oxygen‐related groups into the Ti_3_C_2_T_x_ nanosheets. The work function of oxidized Ti_3_C_2_T_x_ nanosheets (*φ*_sample_) was estimated by measuring the contact potential difference (*V*_CPD_) with KPFM.^[^ ^28^, ^29^ ^]^ Moreover, for the sake of comparison and to gain insight into the mechanism for the work function adjustment, hydrogen plasma treatment (HPT) was adopted as well. The typical surface potential mapping and extracted potential curves of the HPT, OPT, and SPO Ti_3_C_2_T_x_ films with work functions of 4.7, 5.4, and 6.0 eV are shown in **Figure** **2**a–c, respectively. Notably, the reversible tuning of the work function of Ti_3_C_2_T_x_ was realized by successive HPT and OPT (top panel in Figure 2d). Additionally, 30 h SPO treatment of Ti_3_C_2_T_x_ hydrocolloids can raise the work function up to 6.0 eV (bottom panel in Figure 2d). Obviously, the SPO‐Ti_3_C_2_T_x_ nanosheets displayed a higher work function than those treated with OPT due to the larger amount of oxygen‐containing functional groups being introduced onto the surface. In fact the SPO treatment is carried out in an aqueous solution and it enables the oxidation of both surfaces of Ti_3_C_2_T_x_ nanosheets, whereas OPT is performed on previously sprayed nanosheet films supported on a solid substrate, thus enabling the oxidation of a single surface. Significantly, the HPT/OPT and SPO methods have the advantage of bi‐directional and wide‐range (>1 eV) tuning of the work function for Ti_3_C_2_T_x_ nanosheets, thus representing a versatile tool for tailoring optimal contacts with a wide array of semiconductors across a broad energy spectrum when compared to the other chemical modulation methods (Table S1, Supporting Information).

To unravel the mechanism ruling the work function adjustment in Ti_3_C_2_T_x_ nanosheets, Raman and X‐ray photoelectron spectroscopy (XPS) measurements accompanied by density functional theory (DFT) calculations were performed. For the OPT Ti_3_C_2_T_x_ films, we observed an increase in the relative intensity of the Ti_3_C_2_O_2_ Raman peak compared to the untreated Ti_3_C_2_T_x_ nanosheets, indicating the Ti_3_C_2_ oxidation (Figure 2e). The strongest Raman peak intensity of Ti_3_C_2_O_2_ indicates the highest oxidation degree of the SPO Ti_3_C_2_T_x_ nanosheets. Additionally, the relative Raman peak intensity of Ti_3_C_2_(OH)_2_ and Ti_3_C_2_O_2_ increases and decreases respectively after HPT, suggesting the increase of OH groups, transforming from C─O groups.^[^ ^30^, ^31^ ^]^ The XPS results in Figures 2f and S1 (Supporting Information) are in agreement with the findings of Raman spectroscopy.^[^ ^32^, ^33^, ^34^ ^]^ These spectra analyses firmly support that the HPT and OPT (SPO) increase the content in OH and O groups exposed on the Ti_3_C_2_T_x_ nanosheets, respectively.

In principle, the change in work function (∆*W*) of Ti_3_C_2_T_x_ is related to surface dipole moment density (∆*D_s_*):^[^ ^21^ ^]^

(1) $$\Delta W=C\Delta D_{s}+\Delta E_{F}$$

where *C* is a constant and ∆*E_F_* is the Fermi level shift caused by chemical bonding between the terminations and Ti atoms. The ∆*E*_F_ contribution is minimal as its magnitude is below 100 meV.^[^ ^35^, ^36^ ^]^ For Ti_3_C_2_T_x_, the O and OH terminations with positive and negative *D_s_* dominate the charge redistribution thereby controlling the work function changes. To verify the above experimental observation and defined mechanism, DFT calculations were carried out to simulate the work function of the OPT (SPO) and HPT Ti_3_C_2_T_x_ by using monofunctional structures of Ti_3_C_2_O_2_ and Ti_3_C_2_(OH)_2_. The hexagonal close‐packed site marks the surface termination located above the carbon atoms, as shown in Figures 2g,h. The calculated work function of Ti_3_C_2_O_2_ and Ti_3_C_2_(OH)_2_ amounts to 6.1 and 3.2 eV, respectively, being higher and lower than that of Ti_3_C_2_ (Figure S2, Supporting Information). These theoretical results agree with the variation tendency observed in our experiments and verify that the changes in the work function of Ti_3_C_2_T_x_ nanosheets are caused by surface terminations. The theoretically calculated work functions of Ti_3_C_2_O_2_ and Ti_3_C_2_(OH)_2_ exhibit higher and lower values than the experimental data, respectively. This discrepancy is understandable since the theoretical calculations assume the extreme scenario in which the Ti_3_C_2_ surface is fully occupied by O or OH functional groups. In contrast, in the real experimental case, HPT‐ or SPO‐Ti_3_C_2_T_x_ surfaces display a complex mixture of functional groups, including OH, O, and other types, along with vacant sites.

### 2.2 Optoelectronic Performance of High Work Function TA Hybrid Transparent Electrodes

**Figure 3:**
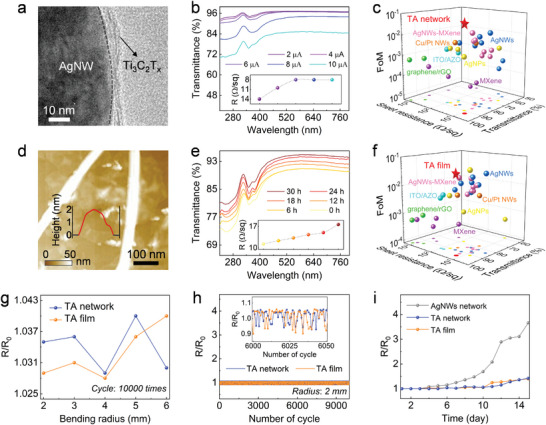
a) TEM image of individual Ti_3_C_2_T_x_ wrapped onto AgNW. b) Variation in optical transmittance of TA networks fabricated by varying electrodeposition current. c) Comparison of the sheet resistance, averaged transmittance in 220–400 nm, and FoM values of the TA networks with other transparent conductors. d) AFM image of Ti_3_C_2_T_x_ nanosheets placed underneath and above the AgNWs network showing the microstructure of TA films. e) TA films fabricated by varying SPO time. Corresponding changes in sheet resistance are shown in the inset. f) Comparison of the sheet resistance, averaged transmittance in 400–800 nm, and FoM values of the TA films with other transparent conductors. g) Relative sheet resistances of the TA networks and TA films as functions of the bending radius. h) Variation of relative resistances of the TA networks and TA films during repeated bending and release at a bending radius of 2 mm. The inset shows the details. i) Variation of relative resistances of the AgNWs networks, TA networks, and TA films in air within 15 days.

The TA networks can be tailored by conformally wrapping the Ti_3_C_2_T_x_ nanosheets onto the AgNWs networks upon using an electrodeposition process we previously developed.^[^ ^37^ ^]^ The as‐formed TA networks exhibit similar surface morphology to AgNWs networks, except that several nanometre‐thick Ti_3_C_2_T_x_ nanosheets are coating the AgNWs (**Figure** **3**a; Figure S3, Supporting Information). The sprayed TA films exhibit a composite sandwich structure made of 1D AgNWs network dressed by top and bottom 2D Ti_3_C_2_T_x_ nanosheet films (Figure 3d). Importantly, the optoelectronic performance of OPT‐TA networks and SPO‐TA films can be optimized by adjusting the electrodeposition current and SPO time of Ti_3_C_2_T_x_ nanosheets, respectively. As portrayed in Figure 3b,e, the optical transmittance of the OPT‐TA and SPO‐TA increases upon decreasing the electrodeposition current and the prolonging time of SPO, while simultaneously the sheets resistance of the OPT‐TA networks and the SPO‐TA films increases. For transparent electrodes, the trade‐off between transmittance and sheet resistance was quantified by estimating the Figure‐of‐merit (FoM),^[^ ^38^, ^39^ ^]^ which is calculated as FoM = *T* ^10^/*R*, where *R* is the sheet resistance, *T* is the average optical transmittance over the measured spectrum range, e.g. 400–800 nm for the TA films and 220–400 nm for the TA networks. A higher FoM value means a better comprehensive optoelectronic performance. The best FoM of OPT‐TA networks and SPO‐TA with average transmittance of 96% (94%) and sheet resistance of 8 Ω sq^−1^ (15 Ω sq^−1^) were obtained when an electrodeposition current of 6 µA and SPO time of 24 h were applied (see Figure S4, Supporting Information for more details). To highlight the optoelectronic performance of the TA hybrids, the sheet resistance, transmittance, and FoM values of diverse transparent conductors were plotted in Figure 3c,f (data listed in Table S2, Supporting Information). Our TA networks exhibit FoM exceeding those of other traditional transparent electrodes and thus represent an alternative electrode type for high‐performance optoelectronic devices. The flexibility and stability of the TA hybrids were further assessed by bending tests and exposure tests in the air. As shown in Figure 3g,h, the TA networks and TA films display sheet resistance changes <5% after 10 000 cycles of repeated bending at various curvature radii (6‐to‐2 mm). The SEM images revealed that both the TA network and the TA film surfaces remained undamaged (Figure S5, Supporting Information). The stability tests of the TA hybrids versus those based on AgNWs networks when exposed to air are portrayed in Figure 3i. The TA hybrids exhibit high stability over a period of 10 days.

### 2.3 OPT‐TA/Ga_2_O_3_ Diodes with Anomalous Schottky Barrier Height

**Figure 4:**
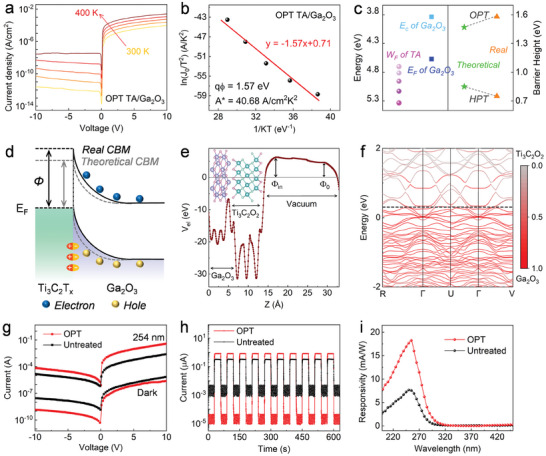
a) Current density‐bias (*J–V*) characteristic curves of the OPT‐TA/Ga_2_O_3_ Schottky diodes recorded at 300, 325, 350, 375, and 400 K. b) The linear relationship between ln(*J*_0_/T^2^) and 1/kT. c) Conduction band minimum (*E*_C_) and Fermi level of the Ga_2_O_3_, the theoretical and real Schottky barrier heights of the OPT and HPT‐TA/Ga_2_O_3_ diodes. d) Electron energy level diagram of the elevated barrier height of OPT‐TA/Ga_2_O_3_ diode. e) Electrostatic potential averaged over planes perpendicular to the Ti_3_C_2_O_2_/Ga_2_O_3_ interface. The zero point in the y‐axis corresponds to Fermi energy. The calculated structure of Ti_3_C_2_O_2_/Ga_2_O_3_ is illustrated in the inset, where purple, pink, dark cyan and light cyan spheres represent Ga, O, Ti, and C atoms, respectively. f) Projected band structures of Ti_3_C_2_O_2_/Ga_2_O_3_. The contribution of Ga_2_O_3_ to the electronic state is colored red, and those of the Ti_3_C_2_O_2_ are colored grey. The dot black lines indicate the approximate location of the Ga_2_O_3_ valence band maximum. g) *I*–*V* characteristic curves, h) current‐time curves of the untreated and OPT‐TA/Ga_2_O_3_ photodetectors, wherein the average photocurrents/dark‐currents are 3.4 × 10^−1^/1.1 × 10^−3^ µA and 8.1 × 10^−1^/1.7 × 10^−5^ µA, respectively. i) spectral responsivity of the untreated and OPT‐TA/Ga_2_O_3_ photodetectors.

The tunable nature of the electric dipole of OPT‐TA makes it possible to control the Schottky barrier height between TA and semiconductors. The TA network was deposited onto a Ga_2_O_3_ film to fabricate a TA/Ga_2_O_3_ diode. Such hybrid film was characterized by SEM, high‐angle annular dark‐field scanning TEM, and energy dispersive spectroscopy (Figure S6, Supporting Information). The Ti_3_C_2_T_x_ nanosheets not only conformally wrapped around the AgNWs but also coated the Ga_2_O_3_ on which AgNWs were previously assembled. The TA/Ga_2_O_3_ Schottky diodes turned out to be suitable device platforms for studying the electrical properties of OPT‐TA and HPT‐TA. In particular, the current density–bias (*J–V*) curves shown in **Figures** **4**a and S7a (Supporting Information) reveal that the OPT and HPT‐TA/Ga_2_O_3_ Schottky diodes are characterized by a clear rectifying behavior. The HPT and OPT‐TA/Ga_2_O_3_ Schottky diodes exhibit room‐temperature rectification ratios of 4.98 × 10^5^ and 4.78 × 10^7^. This large adjustable range of rectification ratio can be ascribed to the difference in Schottky barrier heights.

To quantify the Schottky barrier height of TA/Ga_2_O_3_ diodes, temperature‐dependent *J–V* characteristics were analyzed according to the classical thermionic emission model:^[^ ^40^, ^41^, ^42^ ^]^

(2) $$J=J_{0}\;\left[e^{\left(\frac{qV}{kT}\right)}-1\right]$$

(3) $$J_{0}=AA^{*}\;T^{2}e^{\left(-\frac{q\varphi }{kT}\right)}$$

where *J*_0_ is the saturation current density, *k* is the Boltzmann constant, *T* is the absolute temperature, *A* is the Schottky contact area, and *qφ* is the Schottky barrier height. As the *qφ* is almost constant with temperature, Richardson's plot expresses an approximate linear relationship between ln(*J*_0_/*T* ^2^) and 1/*kT* according to Equation (3). The extracted real barrier height of the OPT and HPT‐TA/Ga_2_O_3_ Schottky junctions amount to 1.57 and 0.75 eV (Figure 4b; Figure S7b, Supporting Information), respectively. Ideally, the Schottky barrier height can be calculated by quantifying the difference between the work function of the electrode and the electron affinity potential of the semiconductor. Toward this end, the band structure of Ga_2_O_3_ was determined by transmission spectrum, XPS valence band spectrum, and KPFM measurements, as shown in Figure S8 (Supporting Information). The Schottky barrier height, calculated according to the energy levels of the Ga_2_O_3_ film and the OPT (HPT)‐TA network, was determined to be 1.47 (0.85) eV, being 0.1 (0.1) eV lower (higher) than the value achieved according to the *J–V* characteristics fitting (Figure 4c). This increase (decrease) of Schottky barrier height can be attributed to the electric dipole effect and agrees with the difference in the dipole moment directions of OPT (HPT) TA networks. In particular, the OPT‐TA with its positive dipole moment generates an electric field pointing toward Ga_2_O_3_. This electric field hinders the electron transfer from TA to the conduction band of Ga_2_O_3_ and reduces the electron affinity potential of Ga_2_O_3_, thus increasing the TA/Ga_2_O_3_ Schottky barrier height, as shown in the energy level diagram (Figure 4d).

To attain further evidence of the effect of surface dipoles on the energy band alignment of Ga_2_O_3_, DFT calculations were carried out on the Ti_3_C_2_O_2_/Ga_2_O_3_ and Ti_3_C_2_(OH)_2_/Ga_2_O_3_ hybrids. Figures 4e and S7c (Supporting Information) show the plane averaged electrostatic potential of the Ti_3_C_2_O_2_/Ga_2_O_3_ and the Ti_3_C_2_(OH)_2_/Ga_2_O_3_. The Φ_0_ and Φ_in_ are the electrostatic potentials of Ga_2_O_3_ before and after contact with Ti_3_C_2_T_x_. The higher Φ_in_ compared to Φ_0_ of Ti_3_C_2_O_2_/Ga_2_O_3_ structure confirms that the dipole direction at the Ti_3_C_2_O_2_/Ga_2_O_3_ interface points from Ti_3_C_2_O_2_ to Ga_2_O_3_ and firmly supports our explanations for the anomalous Ti_3_C_2_O_2_/Ga_2_O_3_ Schottky barrier.^[^ ^43^, ^44^, ^45^ ^]^ To further demonstrate that this strong dipole effect can adjust the interface band alignment through electrostatic interaction, the projected band structures were calculated. We found that the valence band maximum (VBM) of Ga_2_O_3_ in Ti_3_C_2_O_2_/Ga_2_O_3_ exceeds that in Ti_3_C_2_(OH)_2_/Ga_2_O_3_ (Figure 4f; Figure S7d, Supporting Information), indicating the rise of the overall energy band of Ga_2_O_3_ upon interfacing with Ti_3_C_2_O_2_. By and large, these theoretical results confirm the experimentally observed increase of Ti_3_C_2_O_2_/Ga_2_O_3_ Schottky barrier height.

The UV–vis spectrum transparency of the TA networks and the enhanced Schottky barrier height of the TA/Ga_2_O_3_ junction represent a true asset to the performance of self‐powered DUV TA/Ga_2_O_3_ photodetectors (Figure S9, Supporting Information). The current–voltage and current–time curves measured under dark and 254 nm‐light irradiation conditions are displayed in Figure 4g,h. The relationships between photocurrent and light intensity are shown in Figure S10 (Supporting Information). Compared to their untreated counterparts, the photo‐to‐dark current ratio and responsivity of the OPT‐TA/Ga_2_O_3_ devices increased from 310 to 48 000 (Figure 4h) and from 7.57 to 18.24 mA W^−1^ (Figure 4i), respectively. This significant enhancement can be ascribed to the larger Schottky barrier height at the OPT‐TA/Ga_2_O_3_ interface which effectively promotes the separation of photogenerated carriers.^[^ ^46^, ^47^ ^]^ Additionally, a TA/Ga_2_O_3_ photodetector array was fabricated (Figure S11, Supporting Information), which demonstrates the imaging potential of TA electrodes in large‐area flexible (opto‐)electronics.

### 2.4 Optoelectrical Performance of SPO‐TA‐Based OLETs

**Figure 5:**
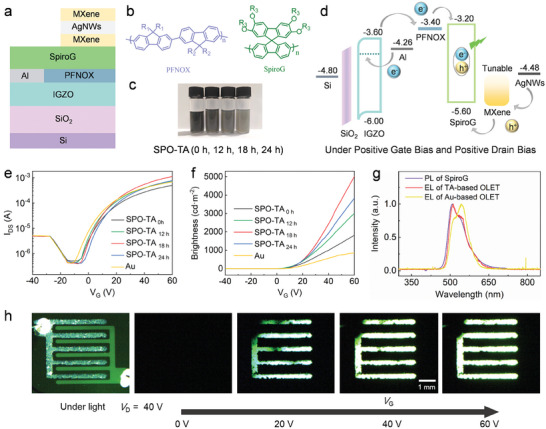
a) Scheme of the OLET architecture with SPO‐TA anode as top electrode. b) Chemical structures of PFNOX and SpiroG. c) Photographs of SPO‐Ti_3_C_2_T_x_ colloidal solutions. d) Energy levels of IGZO, PFNOX, SprioG, and TA. e) Electrical and f) Optical output curves of SPO‐TA‐based OLETs. g) Photoluminescence spectrum of SpiroG, electroluminescence spectra of OPT‐TA‐OLET and Au‐OLET. h) Typical microscopy images of TA‐OLETs with *V*_G_ of 0, 20, 40, and 60 V.

To further prove the versatility of SPO‐TA transparent electrodes in forming Ohmic contact with p‐type organic semiconductors yielding an improvement of the performance of optoelectronic devices, top‐emission OLETs based on various SPO‐TA drain electrodes were fabricated. The device structure consists of multiple vertically‐stacked layers, in which indium‐gallium‐zinc oxide (IGZO), Al, poly[9,9‐bis(60‐(N,Ndiethylamino)hexyl)‐fluorene‐alt‐9,9‐bis(3‐ethyl(oxetane‐3‐ethyoxy)‐hexyl)‐fluorene] (PFNOX), green light‐emitting spiro‐copolymer (SpiroG), SPO Ti_3_C_2_T_x_ nanosheets and AgNWs network operated as the channel layer, source electrode, electron transporting layer, light emissive layer, hole injection layer, and drain electrode, respectively (**Figure** **5**a). The work function of SPO‐TA drain electrode was tuned from 4.89, 5.15, 5.45 to 5.79 eV by varying the solution‐processed oxidization time, named as SPO‐TA_0h_, SPO‐TA_12h_, SPO‐TA_18h_, and SPO‐TA_24h_, respectively. Figure 5b,c displays the chemical structures of soft components and photographs of SPO‐Ti_3_C_2_T_x_ colloidal solutions. Figure 5d shows the energy levels of functional thin films of OLETs. Figure 5e,f illustrates the electrical and optical transfer curves of the OLETs, with applied gate‐source voltages (*V*_GS_) ranging from −40 to 60 V and applied drain‐source voltage (*V*_DS_) of 40 V. All the OLETs present a high current on/off ratio (*I*_On_/*I*_Off_), amounting to ca. 10^3^, and high mobility around 0.2 cm^2^ V^−1^ s^−1^. Notably, the maximum drain‐source current (*I*_D_) and maximum brightness of devices were modified by varying the oxidization time of SPO‐TA. **Table** **1** summarizes the key parameters of all devices. Compared with neat SPO‐TA_0h_ and SPO‐TA_12h_, OLETs based on SPO‐TA_18h_ exhibit a higher maximum drain current of 1.12 mA and a stronger brightness of 5020 cd m^−2^, implying the successful Ohmic contact formed at the interface between SPO‐TA and light emission materials.^[^ ^48^ ^]^ As a result of the prolonged oxidization times, the enhanced work function of SPO‐TA_24h_ determines a decreased maximum *I*_D_ and brightness, which can be attributed to the energy barrier formed again between the Fermi level of SPO‐TA and HOMO level of SpiroG. The programmable nature of the SPO‐TA work function offers huge potential to match the energetics of various organic semiconductors. For the sake of comparison, the electroluminescence (EL) spectra of SPO‐TA_18h_‐based OLETs and the photoluminescence (PL) spectra of the light emission layer were plotted in Figure 5g. A comparable emission peak located at 510 nm with a similar full‐width‐at‐half‐maximum was observed, suggesting the excellent light outcoupling of SPO‐TA. The OLETs with a conventional drain electrode such as Au thin film, were also fabricated for comparison.^[^ ^49^, ^50^ ^]^ Figure 5g reveals the limited performance of Au‐based OLETs as evidenced by a reduction of the maximum brightness down to 856 cd m^−2^ and by a 50 nm red shift of EL emission peak, which can be ascribed to the low transmittance of ≈30% in visible light range (Figure S12, Supporting Information), the light loss from reflectivity of Au thin film. As a preliminary demonstration, SPO‐Ti_3_C_2_T_x_ in solution was patterned, and optical images of a TA‐OLET under gate control were captured, as shown in Figure 5h.

**Table 1:** Comparison of the mobility, maximum drain current (*I*_D_), and maximum brightness of OLETs equipped with different electrodes having various work functions.

Electrode	Work function [eV]	Mobility [cm^2^ V^−1^ s^−1^]	Maximum *I*_D_ [mA]	Maximum brightness [cd m^−2^]
SPO‐TA_0h_	4.89	0.125	0.499	1815
SPO‐TA_12h_	5.15	0.189	0.744	3008
SPO‐TA_18h_	5.45	0.313	1.12	5020
SPO‐TA_24h_	5.79	0.220	0.766	3838
Au	5.20	0.154	0.679	856

## 3 Conclusion

In summary, we developed printable TA hybrids based high work function transparent electrodes making use of two scalable methods, i.e., oxygen plasma treatment and solution‐processed oxidation. The hybrid electrodes revealed simultaneously low sheet resistance (<15 Ω sq^−1^), high transmittance in UV–vis spectrum (>94%), and large range tunable work function (4.7‐to‐6.0 eV). As a proof‐of‐concept application, the printed TA transparent electrodes were integrated into UV sensory and visual display devices. The high work function TA transparent electrodes are capable of establishing Schottky contacts with n‐type semiconductors and Ohmic contacts with p‐type semiconductors, respectively. In the Schottky diode, both experimental and DFT calculation results revealed that surface electric dipoles formed by engineering the functional groups on the MXenes surface are crucial to tailoring an extraordinarily high Schottky barrier exceeding the value resulting from the energy band theory. Moreover, the SPO‐TA‐based top‐emission OLETs display a fourfold enhancement of light emission brightness compared with Au‐based (as conventional high work function noble metal) OLETs, because of the increased visible light transmittance and suitable work function of SPO‐TA films.

The simplicity in the electrode preparation, wide spectral transparency, low sheet resistance, and adjustable work function promote the TA hybrids as multifunctional and versatile transparent electrodes for multiple optoelectronic device applications including perovskite solar cells, organic light‐emitting diodes, and field‐effect transistors. The SPO and OPT methods developed in this study are expected to be a universal approach for adjusting the surface dipole and work function of other MXenes, such as Nb_2_CT_X_ and V_2_CT_x_. Considering the large and rapidly expanding MXenes family, which presently consists of >50 materials with different properties, a variety of novel MXene‐based transparent electrodes can be developed in the future and exploited to fabricate 1D‐2D hybrid structures with programmed work functions by manipulating the MXenes' surface dipole characteristics. Looking ahead, we expect this work will inspire the exploration of more innovative designs and the implementation of further performance enhancements based on diverse MXene‐based optoelectronics and electronics.^[^ ^51^, ^52^, ^53^ ^]^

## 4 Experimental Section

See the Supporting Information.

## Conflict of Interest

The authors declare no conflict of interest.

## Supporting information



Supporting Information

## Data Availability

The data that support the findings of this study are available from the corresponding author upon reasonable request.
