# Supplementary material for: Dressing AgNWs with MXenes Nanosheets: Transparent Printed Electrodes Combining High‐Conductivity and Tunable Work Function for High‐Performance Opto‐Electronics
Source: Adv Mater. 2024 Oct 14;36(48):2412512. doi: 10.1002/adma.202412512 (PMC11602678; doi:10.1002/adma.202412512)
Supplement: Supplementary file 1 — Supporting Information [file ADMA-36-2412512-s001.docx]

Supporting Information

Dressing AgNWs with MXenes nanosheets: transparent printed electrodes combining high-conductivity and tunable work function for high-performance opto-electronics

Zhongshi Ju^⁑^, Yusheng Chen^⁑^, Peng Li*, Jiangang Ma*, Haiyang Xu, Yichun Liu, Paolo Samorì*

Z. Ju, P. Li, J. Ma, H. Xu, Y. Liu

Key Laboratory of UV-Emitting Materials and Technology, Ministry of Education, Northeast Normal University, Changchun, 130024, PR China
E-mail: lip032@nenu.edu.cn (P. Li), majg@nenu.edu.cn (J. Ma)

Y. Chen, P. Samorì
Université de Strasbourg, CNRS, ISIS, 8 allée Gaspard Monge, Strasbourg 67000, France

E-mail: samori@unistra.fr (P. Samorì)

**Experimental Methods**

*Materials:*

Ti_3_C_2_T_x_ colloidal solution and AgNWs colloidal solution were purchased from Nanjing XFNANO Materials Tech Co. Ltd. SpiroG, In(NO_3_)_3_·xH_2_O, Ga(NO_3_)_3_·xH_2_O and Zn(OAc)_2_·2H_2_O were purchased from Sigma-Aldrich. PFNOX was purchased from Luminescence Tech. Corp. All the materials were used as received, without any further purification.

*Preparation of TA films and TA networks:*

The TA films were assembled by spraying coating sub-sequentially Ti_3_C_2_T_x_ colloidal solution in water (0.15 mg mL^-1^), AgNWs colloidal solution in water (0.1 mg mL^-1^) and Ti_3_C_2_T_x_ colloidal solution in water. The spray nozzle of the spraying coating set-up was placed ca. 15 cm away from the sapphire substrate. In this process the sapphire substrate was supported on a hot plate which provided a constant temperature of 160 ℃. The oxidized Ti_3_C_2_T_x_ colloidal solution to be sprayed was prepared by continuously stirring Ti_3_C_2_T_x_ colloidal solution in water (1 mg mL^-1^) for 6-to-30 h on a hot plate at 50 ℃. The XRD results in Figure S14 further proved that Ti_3_C_2_T_x_ phase can be maintained without TiO_2_ phase transformation by heating and stirring for no more than 30 h. The AgNWs networks were prepared by repeating the above steps of spraying, then the TA networks were fabricated by electrodeposition of Ti_3_C_2_T_x_ nanosheets onto the AgNWs networks. During electrodeposition, iridium plate was used as cathode and AgNWs network was employed as anode. Silver pad contact was coated on the one end of AgNWs network to establish external contact. Constant current ranging from 2-to-10 μA was applied with a sourcemeter (Keithley 2461B). The electrodeposition process was carried out for about 3 min at each current. Next, the TA networks were treated by hydrogen and oxygen plasma for different times. The plasma treatment was conducted with a power setting of 200 W, a gas flow rate of 50 sccm, a pressure of 0.15 torr, and a chamber temperature maintained at 25°C. We also verified that TA networks are antioxidant stable, which is due to the protective effect of shell Ti_3_C_2_T_x_ on AgNWs (Figure S15). The TA network prepared by electrodeposition is suitable for inorganic semiconductor devices operating in ultraviolet band. The TA film obtained by two-step spraying is suitable for organic semiconductor devices operating in the visible band, as organic materials are not compatible with the electrodeposition method or the plasma treatment.

*Preparation of IGZO precursor solution, PFNOX solution and SpiroG solution:*

In(NO_3_)_3_·xH_2_O, Ga(NO_3_)_3_·xH_2_O and Zn(OAc)_2_·2H_2_O were dissolved in 2-methoxyethanol at a concentration of 0.2 M in the ratio of 7:1.5:1.5 and stirred at room temperature overnight. 10 mg of PFNOX were dissolved in 5 mL of methanol and 50 μL of acetic acid and then stirred at 80 °C overnight. 80 mg of SpiroG was dissolved in 10 mL of toluene and then stirred at 80 °C overnight.

*Fabrication of TA/Ga_2_O_3_ diodes:*

The Ga_2_O_3_ films were deposited on the sapphire and ITO/polyethylene terephthalate substrates by using radio frequency magnetron sputtering technology at room temperature. The base pressure of the growth chamber was 1 Pa. The sputtering power is 100 W, the sputtering time is 30 mins, and a mixture of argon and oxygen with the flow ratio of 80:1 was introduced in the chamber. Planar Al/Ga_2_O_3_/TA diodes were used to fit the Ga_2_O_3_/TA Schottky barrier height, while vertical ITO/Ga_2_O_3_/TA diodes were employed for photodetection. The TA networks were deposited on the Ga_2_O_3_ substrates by repeating the above steps of spraying and electrodeposition to obtain the TA/Ga_2_O_3_ diodes followed by hydrogen and oxygen plasma treatment for different times.

*Fabrication of OLETs:*

Devices were fabricated on the substrates of n++ Si as bottom gate coated with 230 nm of thermally grown SiO_2_ as the gate dielectric (IPMS Fraunhofer Institute). The IGZO precursor solution was spin-coated at 3000 rpm for 45 s on the top of Si/SiO_2_ substrate, following dried at 150 °C for 5 min and baked at 360 °C for 30 min. Al electrode (50 nm) was evaporated on the IGZO film through shadow masks. Electron transport layer with thickness of 15 nm was spun from PFNOX solution, followed by a thermal annealing at 150 °C for 10 min. The SpiroG layer with thickness of 70 nm was spun from SpiroG solution, followed by thermal annealing at 110 °C for 10 min. For Au-based OLETs, 30 nm Au were deposited through shadow masks. For SPO-TA-based OLETs, SPO-Ti_3_C_2_T_x_, AgNWs and again SPO-Ti_3_C_2_T_x_ were sequentially spray-coated from ethanol solution with concentration of 0.5 mg/mL through shadow masks. The channel width (W) and length (L) were 27 and 0.2 mm, respectively, and the emission area was defined as 4.22 mm^2^.

*Materials characterization and devices test:*

The morphology of the samples were characterized with scanning electron microscopy (SEM, Hitachi S-4800), transmission electron microscope (TEM, JEOL-2100) and atomic force microscopy (AFM, Bruker). High resolution TEM (HRTEM) images were recorded by using an electron microscope FEI Tecnai F20 S-TWIN. High-angle annular dark-field (HAADF) scanning TEM (STEM) investigations were performed by exploiting an electron microscope FEI Talos F200x. Sheet resistances were measured with a four-probe system (RTS-9, Guangzhou) with each value being the average of ten independent measurements. Transmission spectra were measured by a Jasco V-770 spectrometer with an integrating sphere. Work functions were probed with the Kelvin probe force microscopy (KPFM, INNOVA INSP-3). The work function of Ti_3_C_2_T_x_ dried film (*φ*_sample_) was obtained by measuring the contact potential difference (*V*_CPD_) with Kelvin probe force microscope (KPFM) followed by subsequent calculation according to the following Equation (4):

$q\times\Delta V_{CPD}=\varphi_{Au}-\varphi_{sample}$ (4)

where *q* is the elementary charge and *φ*_Au_ is the work function of standard Au film. Structural characterizations were accomplished by X-ray diffraction (XRD) using a Rigaku D/max-2500 set-up. Raman spectra were studied using a Horiba HR evolution spectrophotometer system equipped with a 488 nm excitation source. X-ray photoelectron spectroscopy (XPS) investigations and valence band edge spectrum studies were carried out with Thermo Scientific Escalab 250 Xi. The absorbance was measured by using UV-Vis spectrum equipment (Hitachi UH4150 spectrometer). Current-voltage (I-V) characteristics, spectral responsivity and time-dependent response characteristics were measured by using a probe station equipped with a Xenon lamp, a monochromator and a Keithley 2461B source meter. A Newport 1916-C optical power meter was used to measure the incident light intensities, and the attenuation plate was used to adjust the incident light intensities. OLETs were characterized in a dry and nitrogen-filled glove-box. The electrical characteristics were measured by a Keithley 2636 source meter. Irradiation intensity of device was measured by a photodiode (HAMAMATSU S3204-08) which had been previously calibrated by a luminance meter (KONICA MINOLTA, LS-100).

*Density functional theory calculation methods:*

All density functional theory (DFT) computations were conducted utilizing the Vienna Ab Initio Simulation Package (VASP), adopting the generalized gradient approximation (GGA) with the Perdew-Burke-Ernzerhof (PBE) functional. The projected augmented wave (PAW) method was selected to model the ionic cores, incorporating valence electrons via a plane wave basis with a 450 eV kinetic energy cutoff. The Gaussian smearing technique, with a width of 0.05 eV, was applied to permit partial occupancy of Kohn-Sham orbitals. The electronic energy was deemed self-consistent once the energy variation was less than 10^−4^ eV. The geometry optimization was deemed complete when the change in force dropped below 0.05 eV Å^-1^. For the description of van der Waals interactions, Grimme’s DFT-D3 approach was implemented. Integration over the Brillouin zone was carried out using a Monkhorst-Pack scheme with a k-point mesh of 3 × 3 × 1. DFT calculations were performed on a randomly selected hexagonal close-packed (HCP) site of Ti_3_C_2_T_x_. Our theoretical results, based on the above structural model, indicate that the presence of the O (OH) functional groups can determine an increase (decrease) the work function of Ti_3_C_2_T_x_, in line with the observed trend in face-centered cubic (FCC) sites.**-**

**
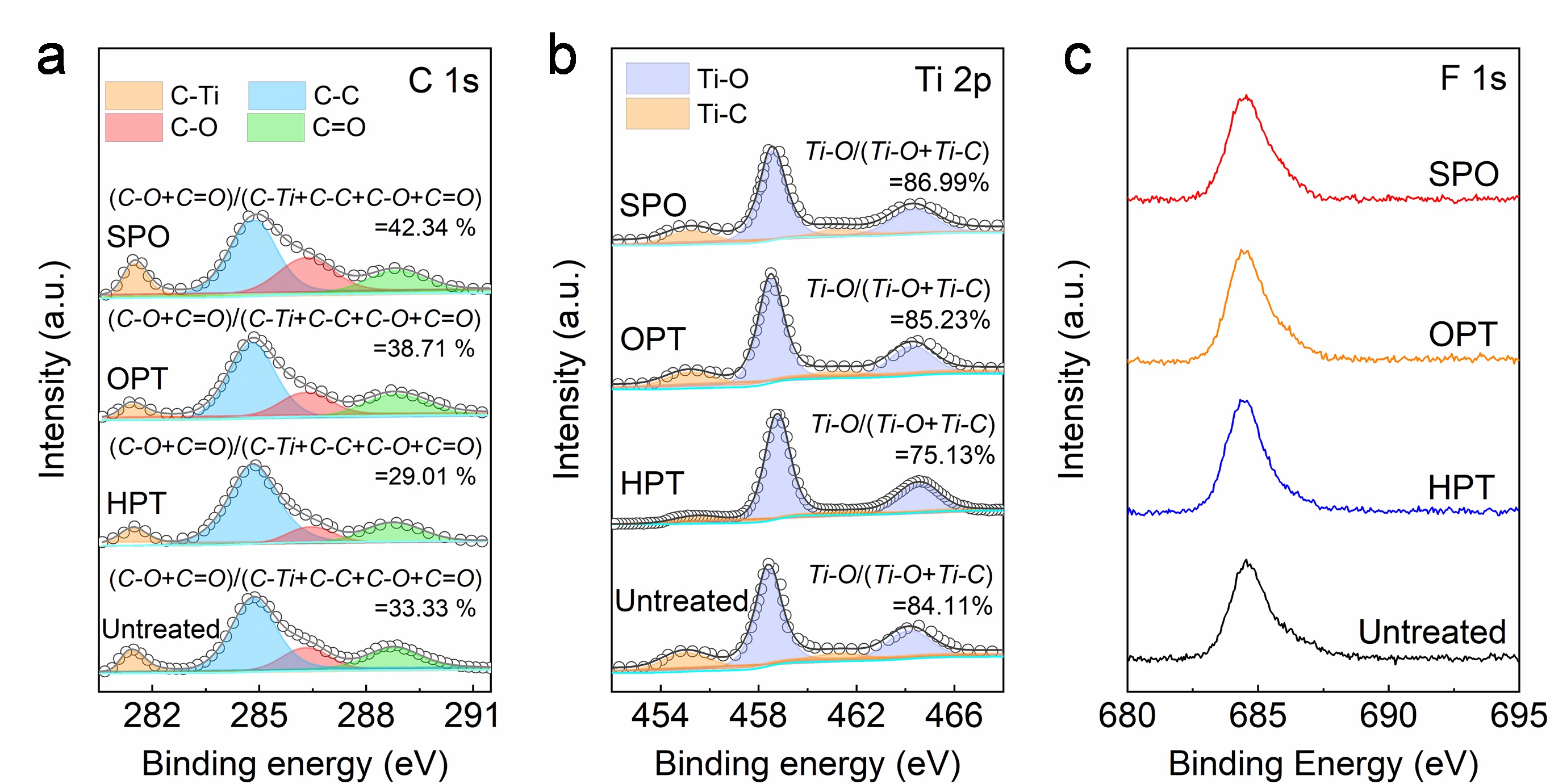
**

**Figure S1.** a) C 1s, b) Ti 2p and c) F 1s XPS spectra of untreated, HPT, OPT and SPO Ti_3_C_2_T_x_.

The XPS results in Figures S1a,b provide unambiguous evidence that HPT reduces the number of O groups, while the OPT and SPO increase the number of O groups. Notably, the oxidation induced by SPO is more intense and more O groups are introduced. Compared with C-O and C=O, the change of Ti-O content in the process of HPT, OPT and SPO is relatively weak. Therefore, we choose a simple model in which the functional group is above the C atom in DFT calculation process. As another important functional group besides O and OH, F groups do not participate in HPT, OPT and SPO processes, the XPS peak of F1s remains unchanged, as shown in Figure S1c.


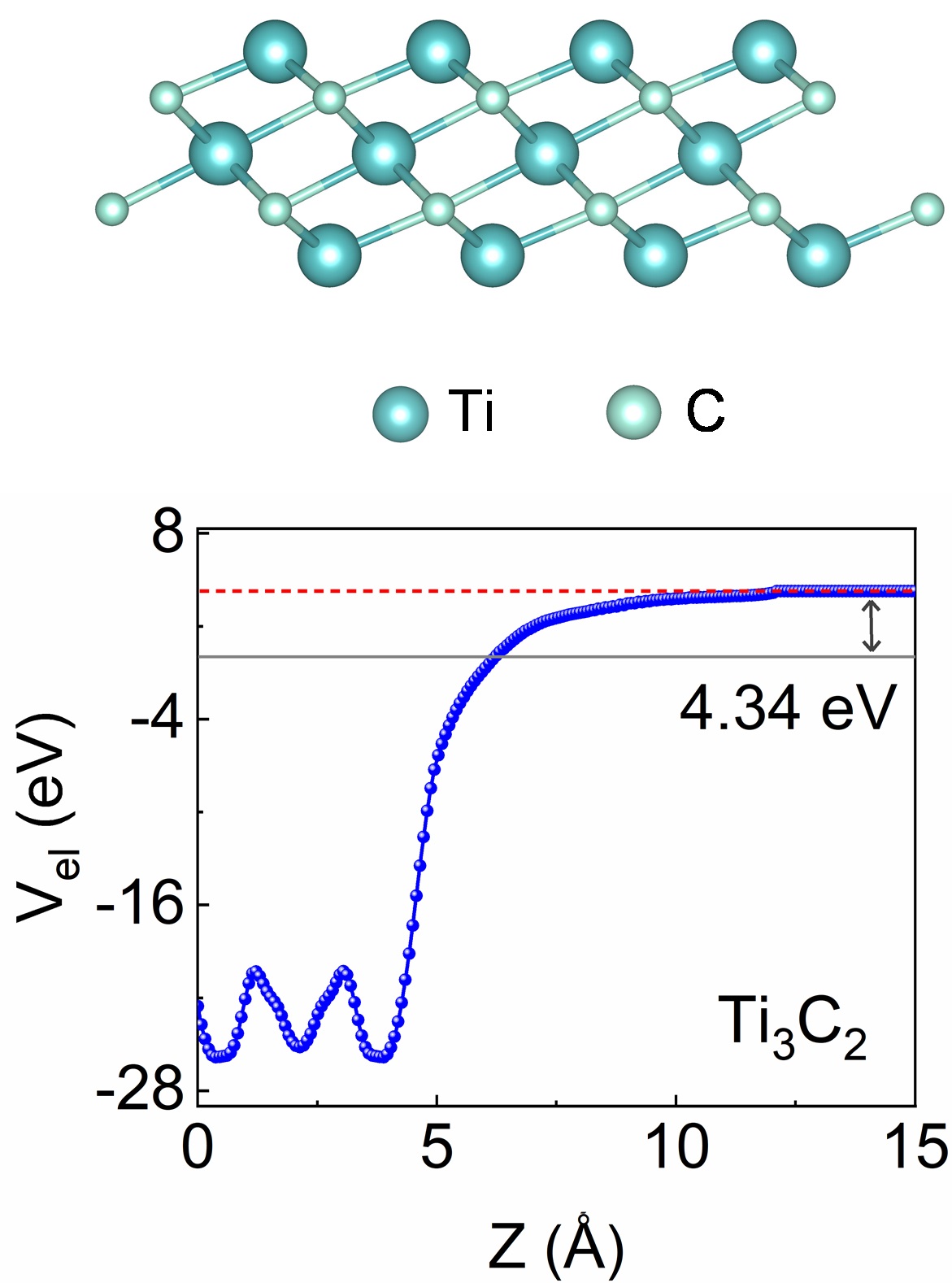


**Figure S2.** Computed structures and DFT-derived plane-averaged electrostatic potentials (V_el_) of Ti_3_C_2_.

According to DFT calculations, the work function of Ti_3_C_2_O_2_, Ti_3_C_2_ and Ti_3_C_2_(OH)_2_ amounts to 5.95 eV, 4.34 eV and 3.15 eV, in good agreement with the experimentally estimated trend values.

**
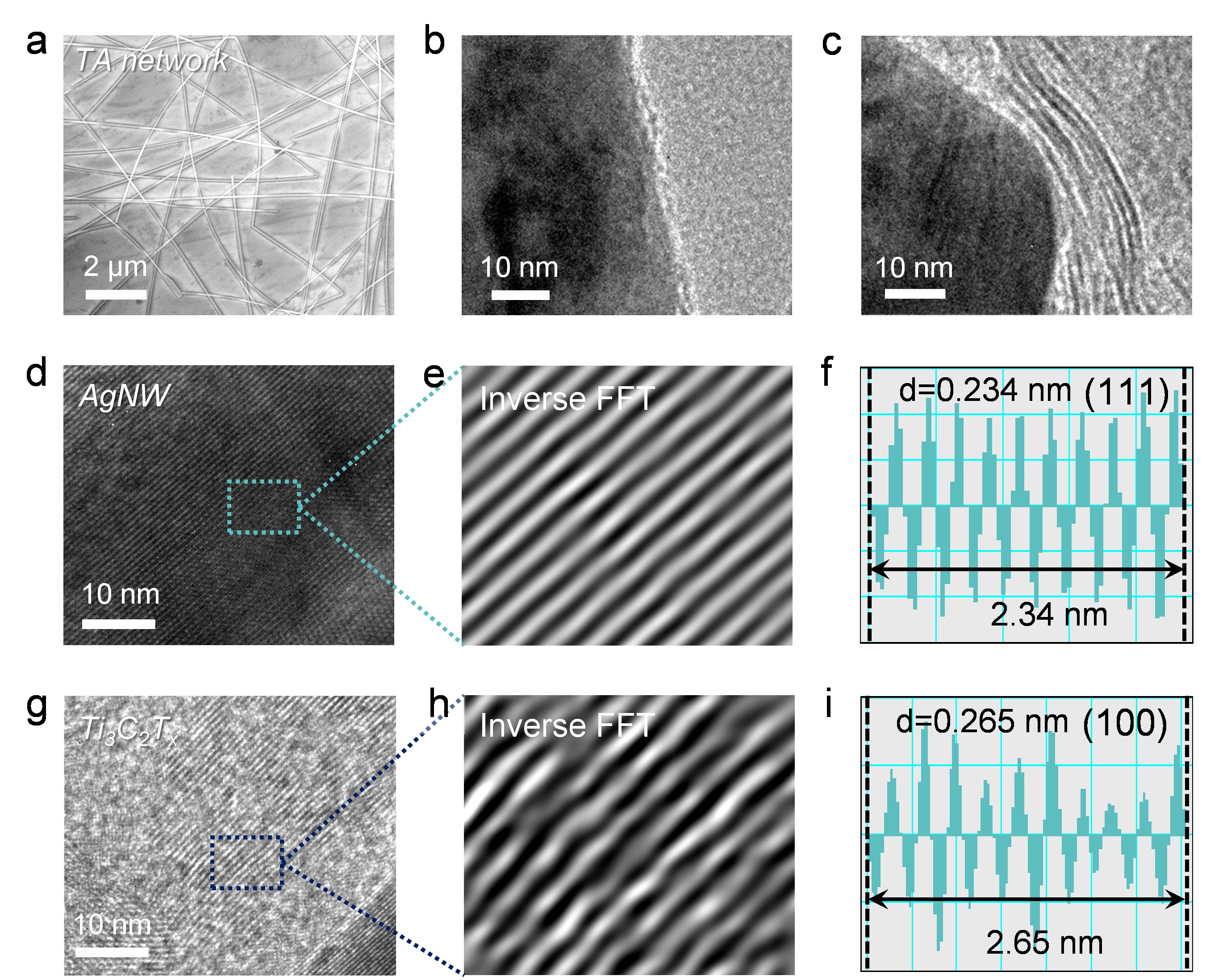
**

**Figure S3.** Microstructure of TA networks. a) SEM image of the TA network. b) and c) HRTEM images of the TA network with different Ti_3_C_2_T_x_ thicknesses. HRTEM images of d) AgNW and g) Ti_3_C_2_T_x_ in the TA network. Inverse fast Fourier transform (FFT) images of e) AgNW and h) Ti_3_C_2_T_x_. Interplanar spacing value of the polycrystalline region of f) AgNW and i) Ti_3_C_2_T_x_.

The topological morphology of the as-prepared TA networks was confirmed by scanning electron microscopy (SEM) and transmission electron microscope (TEM) characterization. Figures S3a-c display that the macroscopic surface morphology of the TA networks is similar to that of the original AgNWs networks except that several nanometer-thick Ti_3_C_2_T_x_ nanosheets are conformally coated onto the AgNWs. The HRTEM images of AgNW and Ti_3_C_2_T_x_ show clear light-dark contrast, and clear lattices are observed (Figures S3d,g). After inverse FFT process of the lattice region (Figures S3e,h), Figures S3f,i show the interplanar spacing values of this polycrystalline region, where the sum of ten interplanar spacings is 2.34 and 2.65 nm, respectively. The interplanar spacings are 0.234 and 0.265 respectively, corresponding to the Ag (111) and Ti_3_C_2_T_x_ (100). During the electrodeposition process, the deposition of Ti_3_C_2_T_x_ nanosheets randomly wraps around the AgNWs. Consequently, the thickness of the Ti_3_C_2_T_x_ nanosheets layers may exhibit variations within the scale of a few nanometers. However, the exceptional electrical conductivity and high transmittance of the TA networks indicate that the micro/nano-scale nonuniformity in the Ti_3_C_2_T_x_ shell has limited detrimental effects. Furthermore, as depicted in Figure S3b, Figure 3a, and Figure S3c, the AgNW networks can be fully encapsulated by the Ti_3_C_2_T_x_ nanosheets, irrespective of the magnitude of the electrodeposition current. The Ti_3_C_2_T_x_ shell thus is capable to enhance the electrical conductivity and to provide a tunable work function to the TA networks.

**
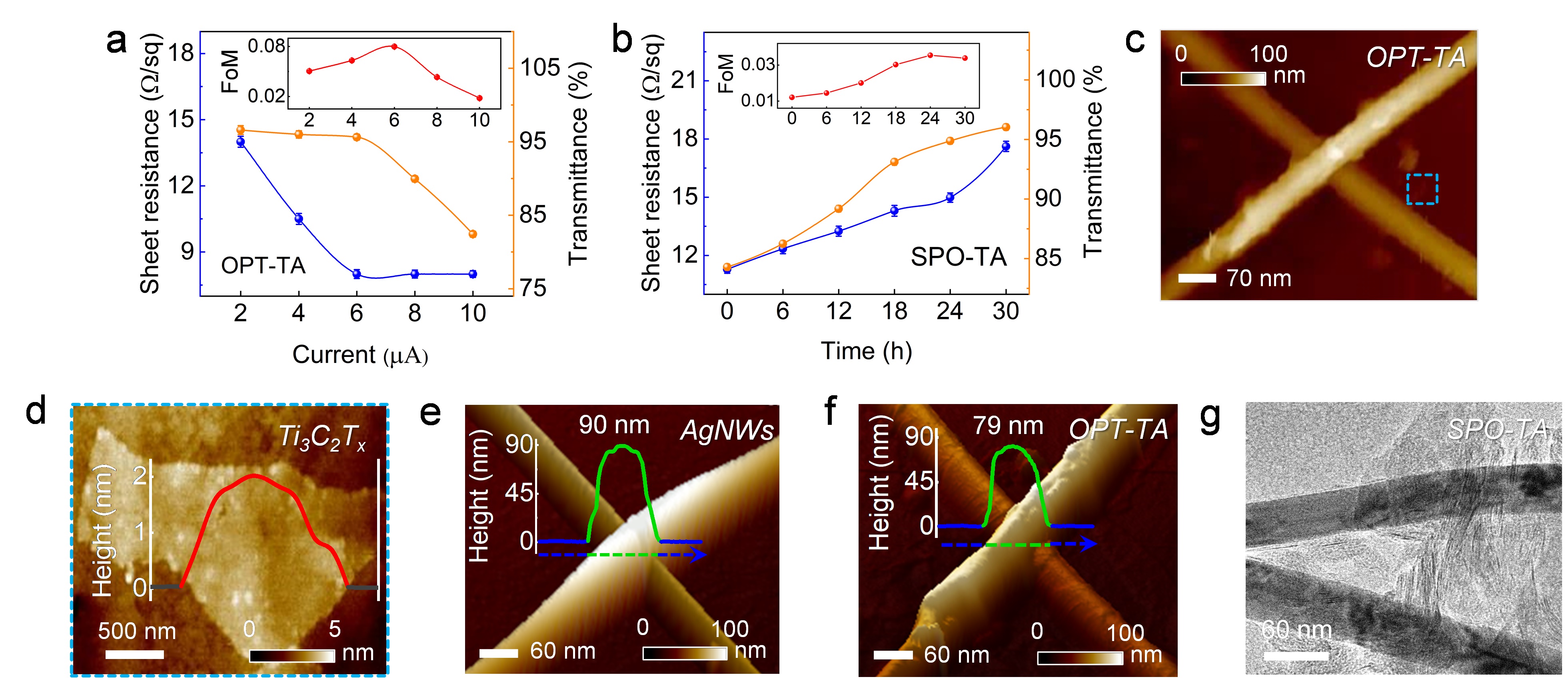
**

**Figure S4.** a) Sheet resistivity, transmittance and FoM of the OPT-TA networks change with the increase of electrodeposition current. b) Sheet resistivity, transmittance and FoM of the SPO-TA films change with the increase of oxidization time. AFM image of the c) OPT-TA network and the d) Ti_3_C_2_T_x_ nanosheet recorded nearby the AgNWs. AFM image of the e) AgNWs network and the f) OPT-TA network. The green line displays the topographical profile of the junction. g) TEM image of the SPO-TA films.

We optimized the optoelectronic performance of the TA networks through the fine-tuning the thickness of the Ti_3_C_2_T_x_ nanosheets by modulating the electrodeposition current. Figure S4a illustrates that, at a constant electrodeposition time, increasing the current to 6 μA led to a gradual decrease in sheet resistance and a corresponding reduction in transmittance, with the FoM value increasing, indicating that a low current (<6 μA) was inadequate to prompt the directed assembly of Ti_3_C_2_T_x_ nanosheets. As the electrodepositing current was further elevated beyond 6 μA, the transmittance decreased, while sheet resistance tended to level off. This could be attributed to an overly aggressive reaction at high currents, causing a rapid and disordered accumulation of Ti_3_C_2_T_x_ nanosheets on the AgNW networks, which failed to form a core-shell structure and did not effectively lower the contact resistance of the AgNWs. In conclusion, the best FoM of OPT-TA networks with average transmittance of 96% and sheet resistance of 8 Ω sq^-1^ were obtained when an electrodeposition current of 6 μA was applied. As depicted in Figure S4b, upon prolonging the time of SPO from 0 to 18 hours, the transmittance, the sheet resistance and FoM of the SPO-TA increased rapidly. Which is because during the early stage of the SPO, untreated Ti_3_C_2_T_x_ had sufficient surface vacancies, allowing a large number of oxygen functional groups to graft onto the Ti_3_C_2_T_x_, resulting in high increasing rates of sheet resistance, transmittance and FoM. By further prolonging the time of SPO from 18 to 24 hours, the surface vacancies on Ti_3_C_2_T_x_ became increasingly saturated, thus the increasing rates were decreased. The best FoM of SPO-TA films with average transmittance of 94% and sheet resistance of 15 Ω sq^-1^ were obtained for a 24 h lasting SPO treatment.

Compared with AgNWs networks, the sheet resistances of TA composites were lower, the decrease in sheet resistance is ascribable to the improved AgNWs contact and extra conduction pathways provided by Ti_3_C_2_T_x_ nanosheets. The AFM images in Figures S4c,d show that the Ti_3_C_2_T_x_ nanosheets exist nearby the AgNWs, and their thickness and diameter are about 2 nm and 1.5 μm, respectively. The height of the overlapped AgNWs decreases after the encapsulation of Ti_3_C_2_T_x_ nanosheets (Figures S4e,f), indicating that the contact between AgNWs became closer. This improved contact could be attributed to the capillary force generated during the solvent evaporation and contributes to reducing the junction resistance of adjacent AgNWs^[1,2]^. Figure S4g demonstrates that the SPO-Ti_3_C_2_T_x_ nanosheets can eliminate the open spaces between the non-connected AgNWs. Large scale SPO-Ti_3_C_2_T_x_ nanosheets layers are covered on the AgNWs, the conductive area is greatly enhanced. Therefore, the conductivities of OPT-TA networks and SPO-TA films were improved compared with AgNWs networks. The design allows conduction bottlenecks of a 1D network (AgNWs) to be circumvented by 2D nanosheets (Ti_3_C_2_T_x_) and vice versa.


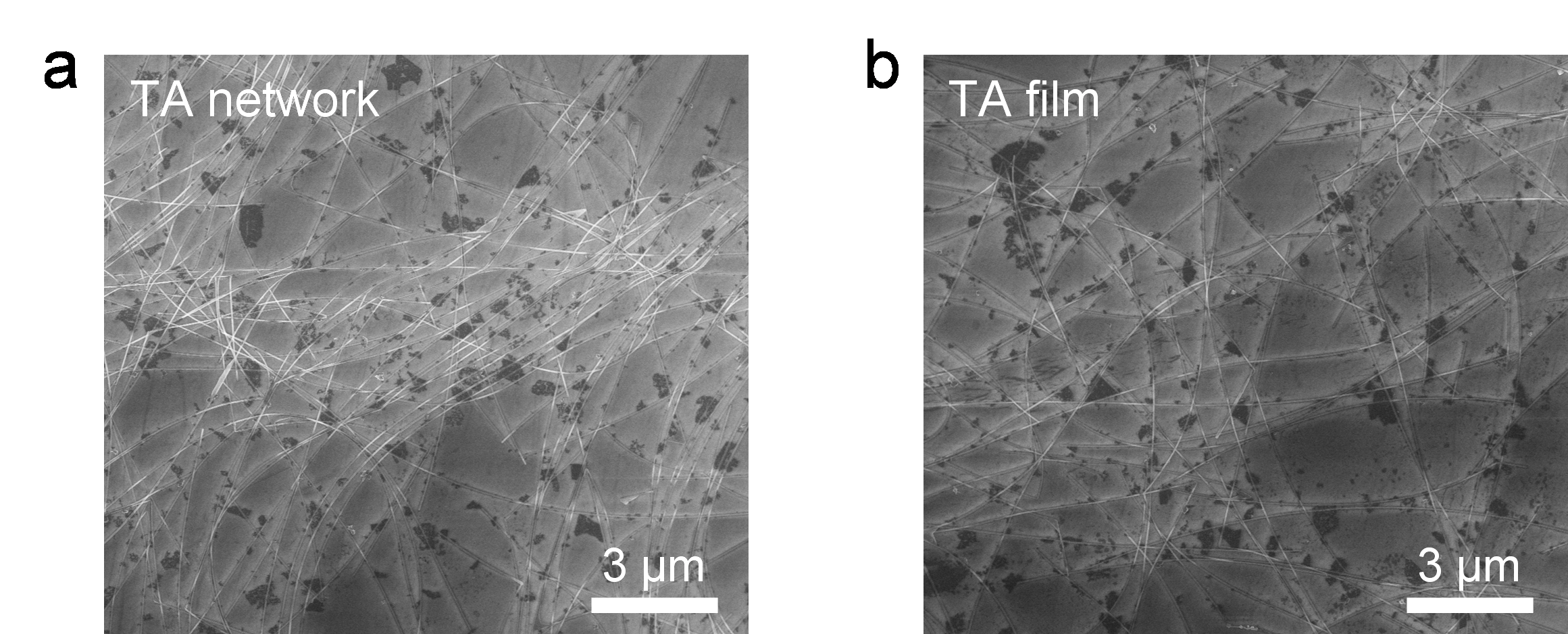


**Figure S5.** SEM images of a) TA network and b) TA film after 10000 cycles of bending and releasing at a bending radius of 2 mm.

The SEM images indicate that the surfaces of both the TA network and the TA film exhibit no apparent fractures or damage after 10000 times of bending and releasing at a bending radius of 2 mm, confirming the excellent flexibility of the TA hybrids.

**
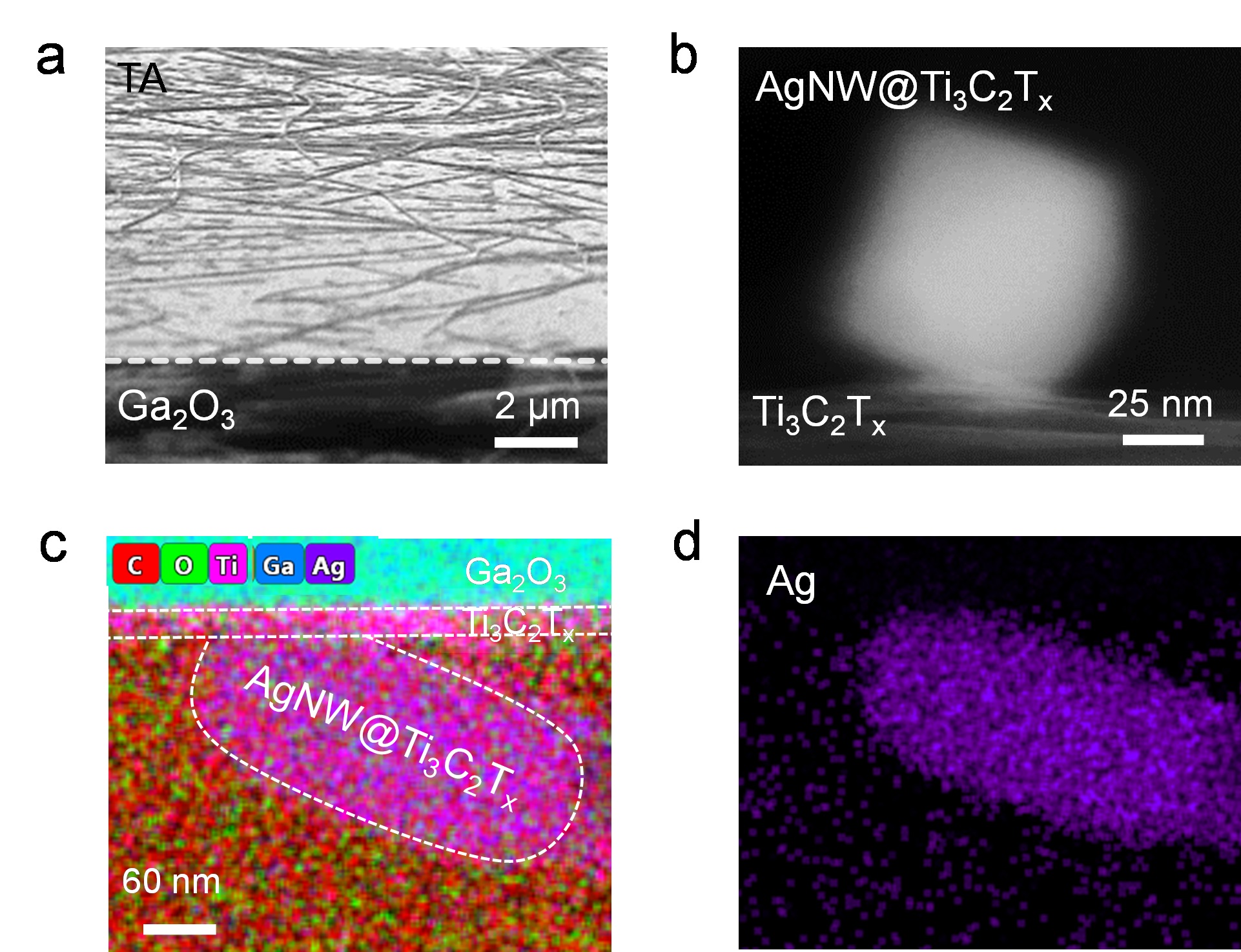
**

**Figure S6.** a) Cross sectional SEM image of the TA/Ga_2_O_3_. b) HAADF STEM image of AgNW@Ti_3_C_2_T_x_. c) and d) HAADF STEM EDS mapping of TA/Ga_2_O_3_.

The tunable electric dipole of TA offers the possibility to control the Schottky height barrier between TA and semiconductors. As a proof-of-concept, we deposited the TA network on the amorphous Ga_2_O_3_ film and constructed a TA/Ga_2_O_3_ diode. The SEM and HAADF STEM image, along with the EDS mapping were combined to record the cross-sectional morphology of the TA/Ga_2_O_3_ heterojunction. The SEM image shows the close contact between TA network and Ga_2_O_3_ (Figure S6a), the HAADF STEM image demonstrates that there are several layers of Ti_3_C_2_T_x_ MXene nanosheets near the AgNWs on the Ga_2_O_3_ (Figure S6b), and the EDS mapping image confirms that the Ti_3_C_2_T_x_ MXene nanosheets not only conformally wrapped on the AgNWs, but also coated on the Ga_2_O_3_ where the AgNWs existed (Figure S6c). As the EDS mapping color of Ti element is overprominent, Figure S6d supplements the EDS mapping of Ag element. The distribution position of Ag and Ti elements is consistent, which confirms the core-shell structure of AgNW@Ti_3_C_2_T_x_. This unique TA/Ga_2_O_3_ heterojunction architecture provides a diode device platform fitting the Schottky characteristic study.

**
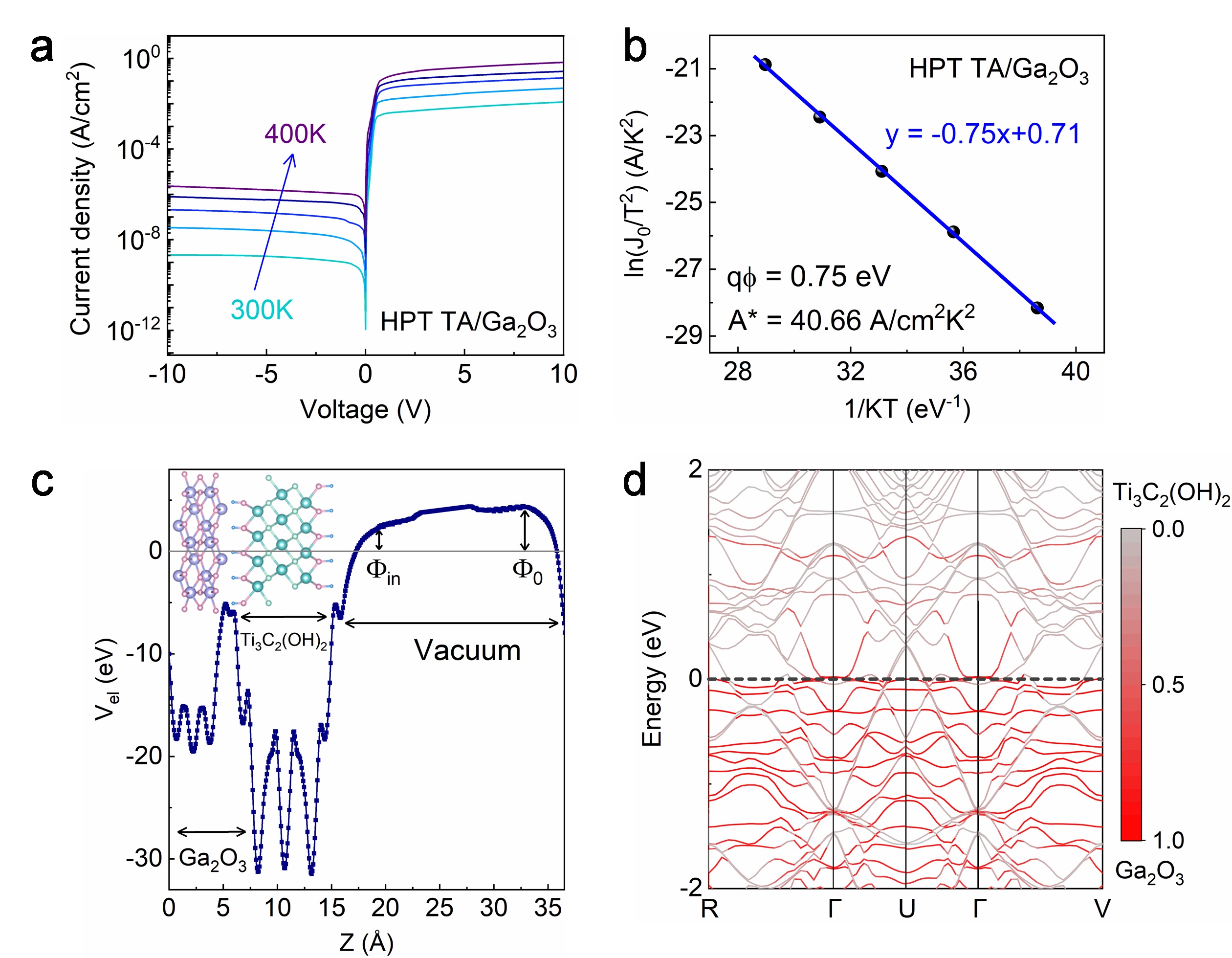
**

**Figure S7.** a) Current density-bias (*J-V*) characteristic curves of the HPT TA/Ga_2_O_3_ Schottky diodes recorded at 300, 325, 350, 375 and 400 K. b) Linear relationship between ln(*J_0_*/T^2^) and 1/kT. c) Electrostatic potential averaged over planes perpendicular to Ti_3_C_2_(OH)_2_/Ga_2_O_3_ interface. The calculated structure of Ti_3_C_2_(OH)_2_/Ga_2_O_3_ is illustrated in the inset, where purple, pink, dark cyan, light cyan and light blue spheres represent Ga, O, Ti, C and H atoms, respectively. d) Projected band structures of Ti_3_C_2_(OH)_2_/Ga_2_O_3_. The contribution of Ga_2_O_3_ to the electronic state is red colored, and those of the Ti_3_C_2_(OH)_2_ are grey colored. The dot black lines indicate the approximate location of Ga_2_O_3_ valence band maximum.

**
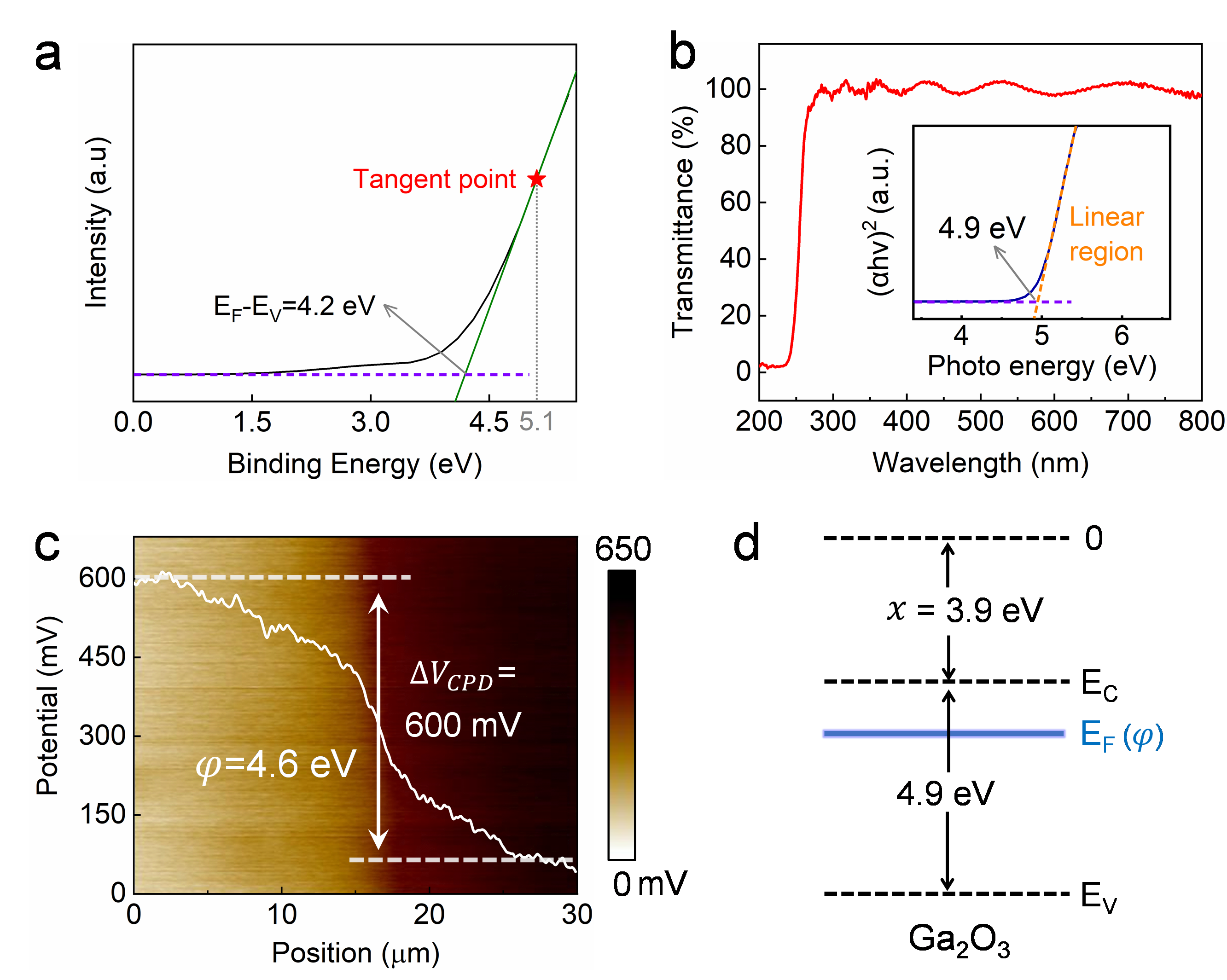
**

**Figure S8.** a) Valence band edge spectrum of Ga_2_O_3_. The tangent point is the extremum of the curve's differentiation, and the value of the VBM is determined by the intersection of the tangent with the background line.^[3-5]^ b) Transmission spectrum of the Ga_2_O_3_ film, with an inset that depicts the relationship between (αhν)² and photon energy. The optical band gap is obtained by extrapolating the linear region and finding its intersection with the background line.^[5,6]^ c) Surface potential distribution recorded on Ga_2_O_3_. d) Schematic diagram of the band arrangement of Ga_2_O_3_.

The energy difference between the valence band maximum (*E*_v_) and the Fermi level (*E*_F_) can be deduced to be 4.2 eV (Figure S8a). The absorption spectrum of Ga_2_O_3_ shows that the optical band gap of Ga_2_O_3_ films is deduced to be 4.9 eV (Figure S8b). The work function of Ga_2_O_3_ is then determined by measuring the contact potential difference. The representative surface potential mapping and the corresponding extracted potential curves for Ga_2_O_3_, with a work function of 4.6 eV, are depicted in Figure S8c. According to the above results, the band arrangement of n-type semiconductor Ga_2_O_3_ is shown in Figure S8d.

**
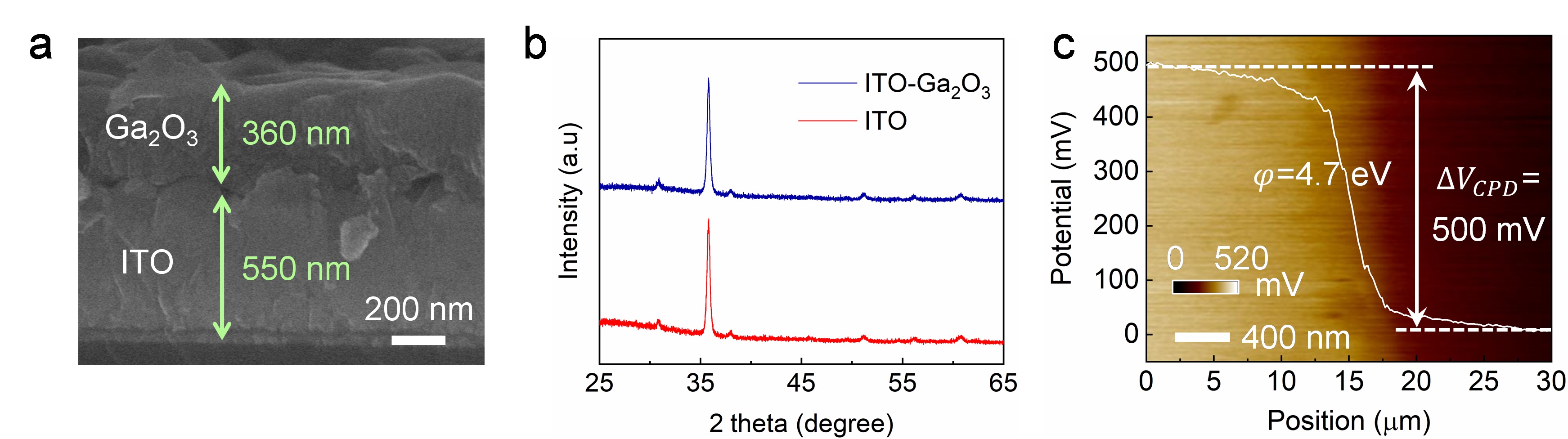
**

**Figure S9.** a) Cross sectional SEM image of Ga_2_O_3_/ITO. b) XRD patterns of Ga_2_O_3_/ITO film and ITO film. c) Surface potential distribution recorded on ITO film.

The Ga_2_O_3_ film was grown on the ITO/PET substrate. The thickness of ITO and Ga_2_O_3_ film is 550 nm and 360 nm, respectively. In the XRD images of the ITO film and Ga_2_O_3_/ITO film display the same distinctive peaks, indicating that the amorphous Ga_2_O_3_ phase forming. The work function of ITO film is 4.7 eV


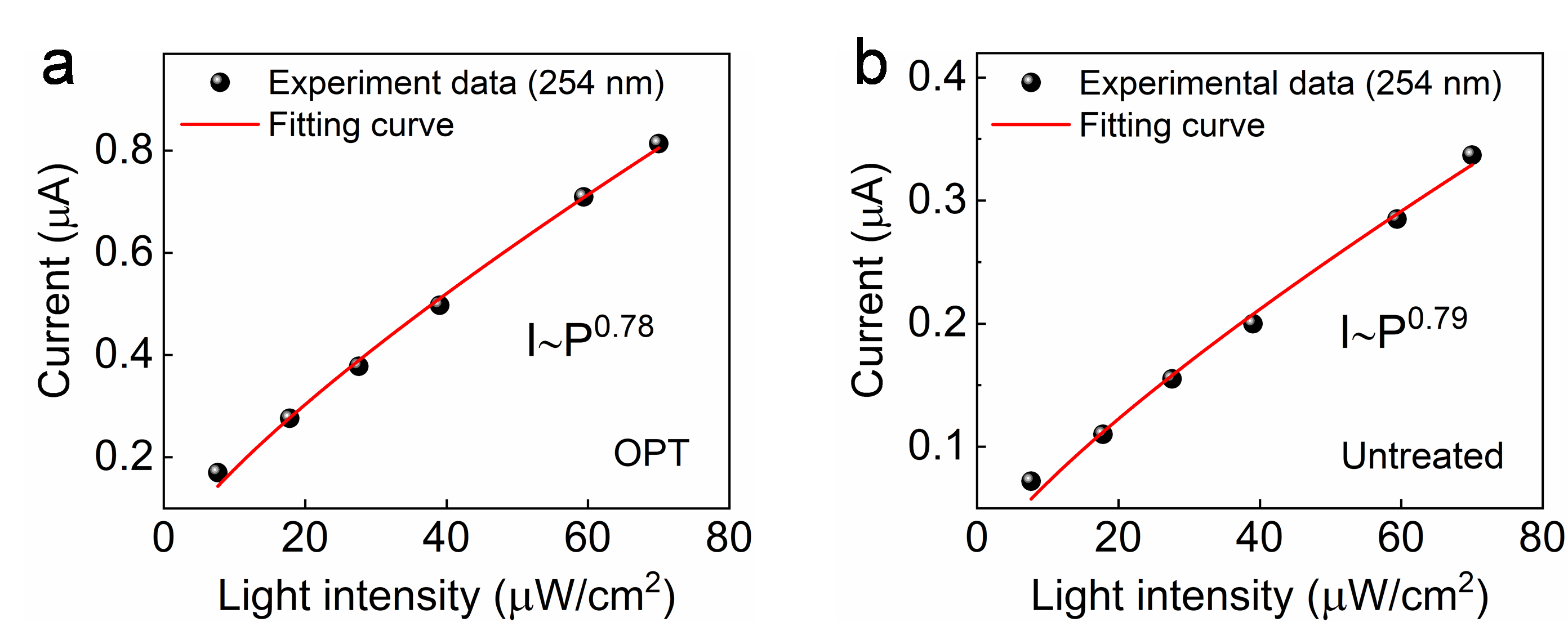


**Figure S10.** Photo current of a) OPT-TA/Ga_2_O_3_ and b) untreated-TA/Ga_2_O_3_ photodetector as a function of light intensity (8-70 μW cm^-2^) under 254 nm illumination fitted by the power law formula.

Figure S10a and b illustrate that the photocurrent is highly dependent on light intensity and increases as the intensity increases. The relationship between photocurrent and light intensity can be described by a power-law equation, I = CP^α^, where I is the photocurrent, C is a constant related to the wavelength of the incident light, P is the light intensity, and α is a fitting factor that represents the photocurrent’s response to light intensity. The calculated α values are 0.79 and 0.78 for the untreated and OPT-TA/Ga_2_O_3_ photodetectors, respectively. The close proximity of these values indicates that the OPT treatment does not significantly affect the surface and trap states of Ga_2_O_3_.


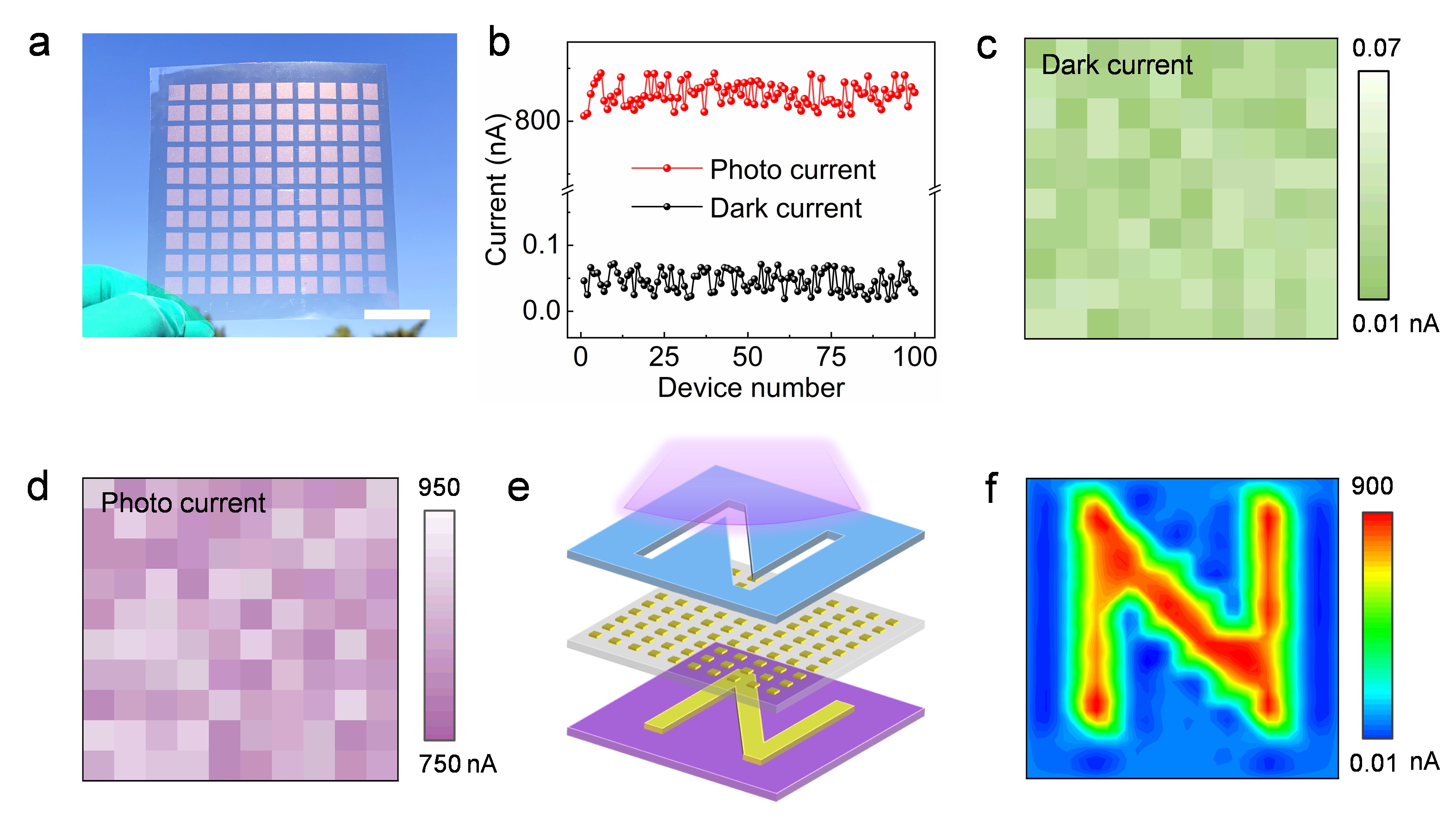


**Figure S11.** a) Digital image of OPT-TA/Ga_2_O_3_ photodetector array, scale bar: 2.5 cm. b) Dark and photo current of all cells in the array. Mapping results of c) dark and d) photo current distributions of all cells in the array. e) Diagrammatic sketch of the imaging system. f) Corresponding 2D mapping result of the customized masks.

The potential application of high work function OPT-TA network transparent electrodes was demonstrated in a large-scale 10 × 10 self-powered OPT-TA/Ga_2_O_3_ photodetector array (Figure S11a). Subsequently, the benefits of room-temperature-deposited OPT-TA/ Ga_2_O_3_ Schottky diodes are demonstrated in the form of photodetector arrays. The uniform distribution of the photo and dark currents across the arrays was confirmed (Figures S11b-d). The small variation range of both the dark current (0.01−0.07 nA) and photo current (756−989 nA) proves the reliability of the arrays and provides a foundation for their large area integration. As shown in Figures S11e,f, a 254-nm light passing through the photomask with hollowed letter “N” can be clearly identified by the current distribution of the OPT-TA/Ga_2_O_3_ photodetector array.

**

**

**Figure S12.** Transmission spectrum of Au film with a thickness of 30 nm.

**

**

**Figure S13.** XRD pattern of Ti_3_C_2_T_x_ after 0, 30 and 36 h solution processed oxidation (SPO).

The diffraction peaks located at 8.5° and 17° correspond to the (002) and (004) crystal faces of Ti_3_C_2_T_x_, respectively. During the first 0 to 30 h, there is no obvious disappearance or shift of these peaks in the Ti_3_C_2_T_x_ diffractograms. Conversely, when the SPO treatment is prolonged to 36 h, the appearance of Ti_5_O_9_ and TiO_2_ derived from oxidation Ti_3_C_2_T_x_ was observed. Thus 30 h is considered being the ultimate stirring time that can be used for introducing the largest amount of O functional groups on the surface of Ti_3_C_2_T_x_. It should be noted that the SPO method requires a precise control of the treatment time, since a long treatment time (36 h) will cause structural transformation or structural degradation of Ti_3_C_2_T_x_.

**
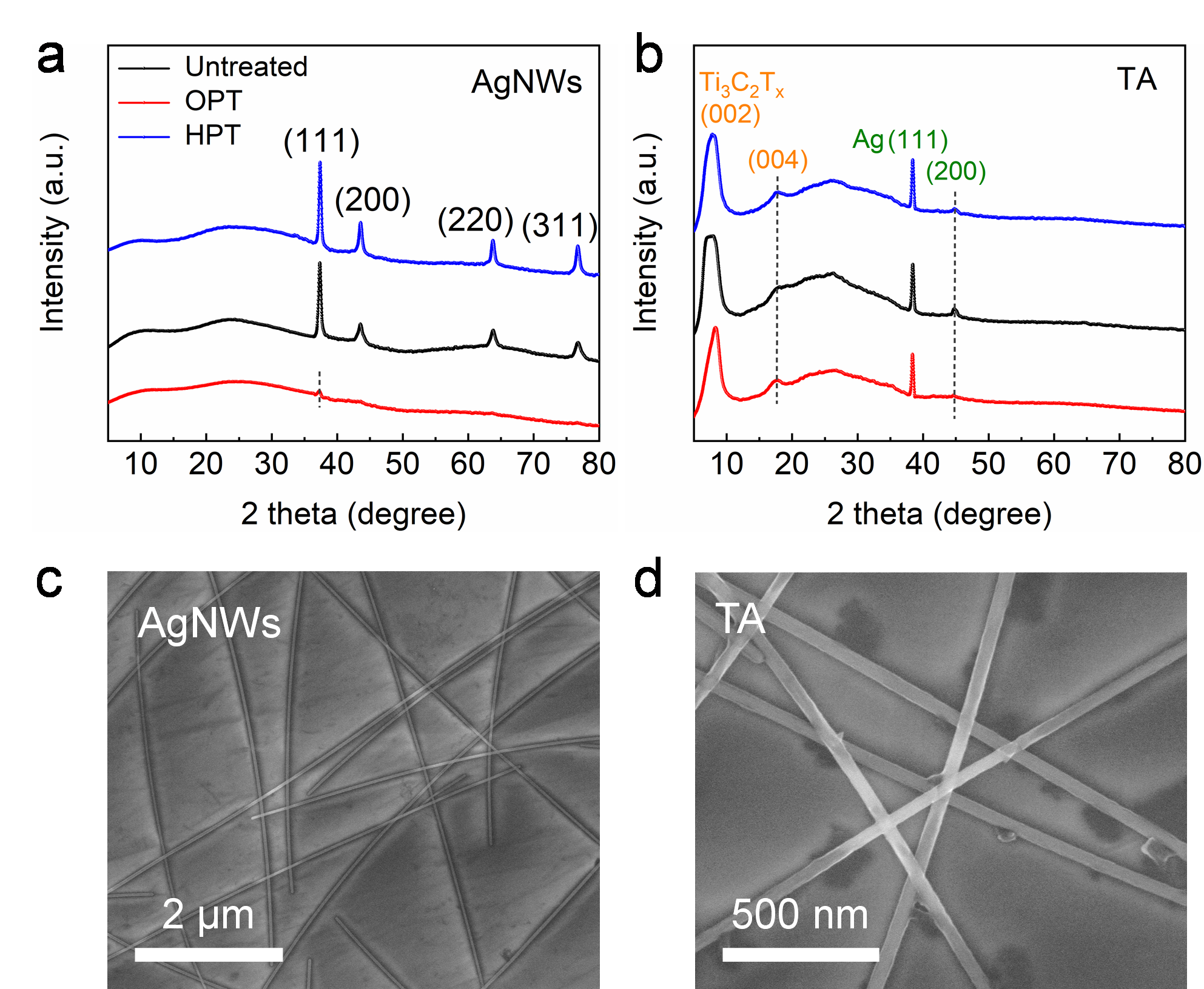
**

**Figure S14.** a) XRD patterns of untreated AgNWs networks, OPT-AgNWs networks and HPT-AgNWs networks. b) XRD patterns of untreated TA networks, OPT-TA networks and HPT-TA networks. c) SEM image of the OPT-AgNWs network. d) SEM image of the OPT-TA network.

In order to test the protective effect of Ti_3_C_2_T_x_ nanosheets on AgNWs, we prepared AgNWs networks and TA networks. In Figure S14a, the XRD peaks of OPT AgNWs almost disappear, proving that OPT can lead to the deterioration of AgNWs. In contrast, the XRD peaks of OPT TA networks are almost the same as untreated TA networks (Figure S14b), demonstrating the protective effect of conformally coating of Ti_3_C_2_T_x_ nanosheets on AgNWs. And the coating of Ti_3_C_2_T_x_ nanosheets has no effect on the network structure of AgNWs (Figures S14c,d).

**Table S1.** Comparison of the changes in the work function of MXenes induced by different modification methods.

| **Method** | **Work function of pristine MXene (eV)** | **Work function of modified MXene (eV)** | **Tuning window (eV)** | **Reference** |
| --- | --- | --- | --- | --- |
| HPT | 4.9 | 4.7 | -0.2 | This work |
| OPT | 4.7 | 5.4 | 0.7 | This work |
| SPO | 4.9 | 6.0 | 1.1 | This work |
| Diazonium covalent modulation | 4.7 | 5.3 | 0.6 | [7] |
| Chemical doping with NH_3_ | 4.9 | 4.4 | -0.5 | [8] |
| Blending DDAB | 5.1 | 4.6 | -0.5 | [9] |
| Surface modification with PEIE/Se | 4.6 | 5.3 | 0.7 | [10] |
| Incorporating S/Cl surface terminations | 3.7 | 4.3 | 0.6 | [11] |

DDAB = didecyldimethyl ammonium bromide

PEIE = polyethylenimine ethoxylated

As shown in Table S1, our OPT and SPO methods demonstrate the greatest capacity for modulating the work function of Ti_3_C_2_T_x_ MXenes compared with previously reported chemical modification techniques. It is important to stress that the optimal electrode choice depends on a comprehensive assessment on its properties of transmittance and conductivity, and also on the work function for modulating the interface resistant. Moreover, raising the work function does not universally enhance the performance of optoelectronic devices. We propose that the most favorable work function value is contingent upon the alignment of the energy bands of the contacting semiconductor. Tailoring the energy band structure to match the positions of the conduction and valence bands, as necessary, is the ideal approach, as evidenced by our OLETs, which exhibit higher brightness when the electrode work function is 5.49 rather than 5.79.

**Table S2.** Comparison of transmittance at visible range of 400-800 nm, transmittance at UV range of 220-400 nm and sheet resistance of our TA network and TA film with other transparent electrodes reported in literature.

| **Material** | **Sheet resistance (Ω sq^-1^)** | **Transmittance at 220-400 nm (%)** | **Transmittance at 400-800 nm (%)** | **Reference** |
| --- | --- | --- | --- | --- |
| TA network | 8.0 | 92.96 | 96.57 | This work |
| TA film | 15.0 | 87.25 | 95.02 | This work |
| AgNWs network | 35.0 | ~85.00 | ~90.00 | [12] |
| AgNWs network | 4.3 | ~78.00 | 83.60 | [13] |
| AgNWs network | 12.0 | ~76.00 | ~86.00 | [14] |
| AgNWs network | 10.0 | ~58.00 | 82.40 | [15] |
| AgNWs mesh | 9.0 | ~80.00 | 87.50 | [16] |
| WO_3_/AgNWs/WO_3_ | 20.0 | ~77.00 | 82.50 | [17] |
| ITO/AgNWs/ITO | 19.6 | ~55.00 | 89.92 | [18] |
| AgNPs | 60.0 | ~82.00 | ~90.00 | [19] |
| AgNPs | 80.0 | ~86.00 | ~78.00 | [20] |
| TiO_2_/AgNPs/TiO_2_ | 7.2 | ~65.00 | 90.97 | [21] |
| MoO_x_/AgNPs/MoO_x_ | 12.1 | ~90.00 | ~59.00 | [22] |
| ITO | 100.0 | ~87.00 | ~93.00 | [23] |
| AZO | 75.0 | ~80.00 | ~93.00 | [24] |
| Cu NWs | 42.0 | ~87.00 | ~87.00 | [25] |
| Pt NWs | 16.0 | ~78.00 | ~82.00 | [26] |
| single layer graphene | 1300.0 | ~98.00 | ~97.00 | [27] |
| bilayer graphene | 600.0 | ~92.00 | ~94.00 | [27] |
| MXene | 1032.0 | ~85.00 | ~95.00 | [28] |
| MXene | 850.0 | ~70.00 | ~85.00 | [29] |
| MXene | 210.0 | ~55.00 | ~75.00 | [30] |
| MXene | 424.0 | ~55.00 | ~87.00 | [31] |
| AgNWs-MXene | 10.1 | ~85.00 | ~87.60 | [32] |
| AgNWs-MXene | 26.0 | 68.54 | 83.32 | [33] |
| AgNWs-MXene | 15.9 | ~80.00 | 92.50 | [34] |
| AgNWs-MXene | 13.9 | ~80.00 | 83.80 | [35] |
| AgNWs-MXene | 17.0 | ~65.00 | 83.20 | [36] |
| AgNWs-MXene | 10.9 | ~68.00 | 82.84 | [37] |
| AgNWs-MXene | 18.0 | ~70.00 | 82.80 | [38] |

**References**

[1] F. Duan, W. Li, G. Wang, C. Weng, H. Jin, H. Zhang, Z. Zhang, *Nano Res*. **2019**, *12*, 1571.

[2] Y. Yang, S. Chen, W. Li, P. Li, J. Ma, B. Li, X. Zhao, Z. Ju, H. Chang, L. Xiao, H. Xu, Y. Liu, *ACS Nano* **2020**, *14*, 8754.

[3] E. A. Kraut, R. W. Grant, J. R. Waldrop, S. P. Kowalczyk, *Phys. Rev. Lett.* **1980**, *44*, 1620.

[4] E. A. Kraut, R. W. Grant, J. R. Waldrop, S. P. Kowalczyk, *Phys. Rev. B* **1983**, *28*, 1965.

[5] J. Huang, C. You, B. Wu, Y. Wang, Z. Zhang, X. Zhang, C. Liu, N. Huang, Z. Zheng, T. Wu, S. Kiravittaya, Y. Mei, G. Huang, *Light Sci. Appl.* **2024**, *13*, 153.

[6] J. Tauc, R. Grigorovici, A. Vancu, *Phys. Status Solidi B* **1966**, *15*, 627.

[7] H.  Jing, H.  Yeo, B.  Lyu, J.  Ryou, S.  Choi, J. H.  Park, B. H.  Lee, Y. H. Kim, S. Lee, *ACS Nano* **2021**, *15*, 1388.

[8] B.  Lyu, M.  Kim, H.  Jing, J.  Kang, C.  Qian, S.  Lee, J. H.  Cho, *ACS Nano* **2019**, *13*, 11392.

[9] H.  Wang, Y.  Wang, Z.  Ni, N.  Turetta, S. M.  Gali, H.  Peng, Y.  Yao, Y. Chen, I. Janica, D. Beljonne, W. Hu, A. Ciesielski, P. Samori, *Adv. Mater.* **2021**, *33*, 2008215.

[10] J. Chen, X. Liu, Z. Li, F. Cao, X. Lu, X. Fang, *Adv. Funct. Mater.* **2022**, *32*, 2201066.

[11] V. Kamysbayev, A. S. Filatov, H. Hu, X. Rui, F. Lagunas, D. Wang, R. F. Klie, D. V. Talapin, *Science* **2020**, *369*, 979.

[12] H. Fang, C. Zheng, L. Wu, Y. Li, J. Cai, M. Hu, X. Fang, R. Ma, Q. Wang, H. Wang, *Adv. Funct. Mater*. **2019**, *29*, 1809013.

[13] O. Ergun, S. Coskun, Y. Yusufoglu, H. E. Unalan, *Nanotechnology* **2016**, *27*, 445708.

[14] F. Oytun, O. Alpturk, F. Basarir, *Mater. Res. Bull*. **2019**, *112*, 53.

[15] S. Jun, K. W. Choi, K.-S. Kim, D. U. Kim, C.-J. Lee, C. J. Han, C.-R. Lee, B.-K. Ju, J.-W. Kim, *Compos. Sci. Technol*. **2019**, *182*, 107773.

[16] Y. Liu, S. Shen, J. Hu, L. Chen, *Opt. Express.* **2016***, 24*, 25774.

[17] Z. Qi, J. Cao, L. Ding, J. Wang, *Appl. Phys. Lett.* **2015**, *106*, 053304.

[18] C. Kim, M. J. Lee, S. J. Hong, Y. S. Kim, J. Y. Lee, *Compos. Sci. Technol*. **2018**, *157*, 107.

[19] J. S. Park, Y. Song, D. Park, Y. W. Kim, Y. J. Kim, *Nanotechnology* **2018**, *29*, 255302.

[20] [J. Shang](https://onlinelibrary.wiley.com/authored-by/Shang/Jun), [H. Qi](https://onlinelibrary.wiley.com/authored-by/Qi/Hongfei), [H. Feng](https://onlinelibrary.wiley.com/authored-by/Feng/Haifeng), [L. Wang](https://onlinelibrary.wiley.com/authored-by/Wang/Liang), [J. Zhang](https://onlinelibrary.wiley.com/authored-by/Zhang/Junying), [Y. Wang](https://onlinelibrary.wiley.com/authored-by/Wang/Yigang), [W. Hao](https://onlinelibrary.wiley.com/authored-by/Hao/Weichang), [T. Wang](https://onlinelibrary.wiley.com/authored-by/Wang/Tianmin), *Staus Solidi RRL*. **2013**, *7*, 1071.

[21] M.-Q. Zhu, H.-D. Jin, P.-Q. Bi, F.-J. Zong, J. Ma, X.-T. Hao, *Appl. Phys*. **2016**, *49*, 115108.

[22] X. Wang, H. Wang, D. Zhou, H. Jin, J. Yu, *Mater. Lett*. **2018**, *230*, 289.

[23] H. D. Kim, M. J. Yun, S. J. Kim, *Alloys Compd.* **2015**, *653*, 534.

[24] [Q. Nian](javascript:;), [M. Callahan](javascript:;), [D. Look](javascript:;), [H. Efstathiadis](javascript:;), [J. Bailey](javascript:;), [G. J. Cheng](javascript:;), *APL Mater*. **2015**, *3*, 062803.

[25] Q. Lonne, J. Endrino, Z. Huang, *Nanoscale Res. Lett*. **2017**, *12*, 577.

[26] Y. Wang, M. Shahid, J. Cheng, H. Nishijima, W. Pan, *Nanotechnology*. **2017**, *28*, 155202.

[27] K. S. Exner, *ACS* *Appl. Energy Mater.* **2019**, *2*, 7991.

[28] [C. Zhang](https://onlinelibrary.wiley.com/authored-by/Zhang/Chuanfang+(John)), [B. Anasori](https://onlinelibrary.wiley.com/authored-by/Anasori/Babak), [A. Seral-Ascaso](https://onlinelibrary.wiley.com/authored-by/Seral%E2%80%90Ascaso/Andr%C3%A9s), [S.-H. Park](https://onlinelibrary.wiley.com/authored-by/Park/Sang%E2%80%90Hoon), [N. McEvoy](https://onlinelibrary.wiley.com/authored-by/McEvoy/Niall), [A. Shmeliov](https://onlinelibrary.wiley.com/authored-by/Shmeliov/Aleksey), [Ge. S. Duesberg](https://onlinelibrary.wiley.com/authored-by/Duesberg/Georg+S.), [J. N. Coleman](https://onlinelibrary.wiley.com/authored-by/Coleman/Jonathan+N.), [Y. Gogotsi](https://onlinelibrary.wiley.com/authored-by/Gogotsi/Yury), [V. Nicolosi](https://onlinelibrary.wiley.com/authored-by/Nicolosi/Valeria), *Adv. Mater*. **2017**, *29*, 1702678.

[29] H. S. Lim, J. M. Oh, J. W. Kim, *ACS Appl. Mater. Interfaces*. **2021**, *13*, 25400.

[30] S. Lee, E. H. Kim, S. Yu, H. Kim, C. Park, S. W. Lee, H. Han, W. Jin, K. Lee, C. E. Lee, J. Jang, C. M. Koo, C. Park, *ACS Nano* **2021**, *15*, 8940.

[31] S. Kumar, D. Kang, V. H. Nguyen, N. Nasir, H. Hong, M. Kim, D. C. Nguyen, Y. Lee, N. Lee, Y. Seo, *ACS Appl. Mater. Interfaces* **2021**, *13*, 40976.

[32] J. Liu, L. Zhang, N. Wang, C. Li, *Nano Energy* **2020**, *78*, 105385.

[33] H. Tang, H. Feng, H. Wang, X. Wan, J. Liang, Y. Chen, *ACS Appl. Mater. Interfaces* **2019**, *11***,** 25330.

[34] J. Liu, L. Zhang, C. Li, *Ind. Eng. Chem. Res.* **2019**, *58*, 21485.

[35] W. Jiang, S. Lee, K. Zhao, K. Lee, H. Han, J. Oh, H. Lee, H. Kim, C. M. Koo, C. Park, *ACS Nano* **2022**, *16***,** 9203.

[36] P. Wang, C. Zhang, M. Wu, J. Zhang, X. Ling, L. Yang, *Nanomaterials* **2021**, *11*, 1360.

[37] [W. Chen](https://pubs.rsc.org/en/results?searchtext=Author:Weipeng%20Chen), [R. Zhang](https://pubs.rsc.org/en/results?searchtext=Author:Ruijia%20Zhang), [X. Yang](https://pubs.rsc.org/en/results?searchtext=Author:Xia%20Yang), [H. Wang](https://pubs.rsc.org/en/results?searchtext=Author:Hongyu%20Wang), [H. Yang](https://pubs.rsc.org/en/results?searchtext=Author:Hanjun%20Yang), [X. Hu](https://pubs.rsc.org/en/results?searchtext=Author:Xiaotian%20Hu), [S. Zhang](https://pubs.rsc.org/en/results?searchtext=Author:Shaohua%20Zhang), *J.* *Mater. Chem. C*. **2022**, *10*, 8625.

[38] [Z. Wang](https://pubs.rsc.org/en/results?searchtext=Author:Zhuochao%20Wang), [P. Wang](https://pubs.rsc.org/en/results?searchtext=Author:Peng%20Wang), [W. Cao](https://pubs.rsc.org/en/results?searchtext=Author:Wenxin%20Cao), [C. Sun](https://pubs.rsc.org/en/results?searchtext=Author:Chunqiang%20Sun), [Z. Song](https://pubs.rsc.org/en/results?searchtext=Author:Zicheng%20Song), [D. Ji](https://pubs.rsc.org/en/results?searchtext=Author:Dongchao%20Ji), [L. Yang](https://pubs.rsc.org/en/results?searchtext=Author:Lei%20Yang), [J. Han](https://pubs.rsc.org/en/results?searchtext=Author:Jiecai%20Han)a, [J. Zhu](https://pubs.rsc.org/en/results?searchtext=Author:Jiaqi%20Zhu), *J. Mater. Chem. C.* **2022**, *10*, 17066.
